# Conductive Polymers in Lithium-Ion Battery Cathodes: Enhancing Performance and Stability

**DOI:** 10.3390/polym18040429

**Published:** 2026-02-08

**Authors:** Mobinul Islam, Gazi A. K. M. Rafiqul Bari, Kyung-Wan Nam

**Affiliations:** 1 Department of Energy & Materials Engineering, Dongguk University, Pildong-ro 1-gil, Jung-gu, Seoul 04620, Republic of Korea; mobinsust@dongguk.edu; 2 Department of Mechanical Engineering, Gachon University, Sujeong-gu, Seongnam-si 13120, Republic of Korea

**Keywords:** polymer composite, cathode material, Li-ion battery, conductive polymer, electronic conductivity

## Abstract

Conducting polymers are extensively researched due to their remarkable characteristics, including adaptable and tunable electrical conductivity, straightforward manufacturing process, enhanced mechanical strength, lightweight composition, affordability, and convenience in handling. Furthermore, they demonstrate outstanding chemical stability and elevated thermal conductivity. Conducting polymers can be easily modified with other substances to create composites with improved performance. They are easy to integrate with other materials, such as metal oxide, metal sulfide, and metal nitride. In the energy sector, especially concerning electrochemical energy storage systems such as lithium-ion batteries (LIBs), conducting polymer composites with metal oxide, phosphate, and sulfide have shown considerable promise as electrode materials. These composites leverage synergistic effects that improve electrical conductivity, increase surface area, and prevent electrode pulverization, ultimately resulting in faster charge and discharge cycles, higher energy density, and enhanced cycling performance. This review offers a comprehensive examination of different types of conducting polymers used in LIB electrodes, provides hints regarding several synthesis methods for producing conducting polymer composites with metal oxide, phosphate, and sulfide, and discusses how the morphology of these composites influences their electrochemical performance.

## 1. Introduction

In stationary systems and transportation applications, the widespread use of petroleum-based sources is associated with concerns such as global warming and the depletion of fossil fuel reserves. These concerns have led to increased interest in alternative renewable energy sources, such as solar, wind, and biofuels [1,2,3]. However, the intermittent nature of renewable energies, such as solar and wind, makes them hard to supply during off-peak periods. The process of renewable energy production is highly unpredictable; therefore, this results in the need for different types of energy storage devices. Energy storage technologies play a vital role in managing energy demand by storing excess energy during periods of energy source availability (e.g., sunlight, wind) and releasing it during high-demand periods [4,5]. Energy storage systems have undergone significant research and development efforts to improve their performance. Technologies such as fuel cells, batteries, supercapacitors, pumped hydro storage, and thermal energy storage are being explored to address the varied needs of different applications [6,7]. Among them, batteries stand out for their high energy density and scalability. They encompass a diverse range of chemistries, including lithium-ion batteries (LIBs), sodium-ion batteries (SIBs), potassium-ion batteries (PIBs), magnesium-ion batteries (MIBs), zinc-ion batteries (ZIBs), and aluminum-ion batteries (AIBs) [8,9,10,11,12,13,14]. Each type of metal-ion battery offers unique benefits in terms of material availability, safety, and affordability.

Lithium-ion batteries (LIBs) are widely used in power tools, consumer electronics, and electric vehicles (EVs) among advanced energy storage technologies. Li-ions offer advantages over other elements for rechargeable batteries, such as a low reduction potential, a small charge-to-ionic radii ratio, and high ion mobility [15,16], which allow LIBs to achieve relatively high volumetric and gravimetric capacities. These characteristics allow them to store more energy in compact sizes and make them ideal for portable electronics and electric vehicles. Other than advantages, LIBs suffer from several issues, such as inadequate ion-transport, electrode instability, and challenges at the electrode–electrolyte interface [17]. The electrode–electrolyte interface in LIBs presents several critical challenges that significantly impact battery performance, safety, and lifespan. One of the main problems is the formation and stability of the solid electrolyte interphase (SEI) on the anode, which is essential for preventing continuous electrolyte decomposition but can degrade over time, leading to capacity fade [18,19]. Similarly, on the cathode side, electrolyte oxidation and transition metal dissolution can result in interfacial instability [20,21]. Volume changes during charge–discharge cycles can cause mechanical stress, cracking, and loss of interfacial contact, especially in high-capacity electrode materials [22]. Addressing these challenges requires advancements in material design, electrolyte formulation, and interface engineering to enhance the overall efficiency and reliability of lithium-ion batteries. This can be achieved through the development of advanced materials with enhanced ion conductivity, the optimization of electrode structures for enhanced stability, and the design of composite electrodes or protective coatings on electrodes to mitigate interface degradation [16,23]. These innovations are crucial to unlocking the full potential of metal-ion batteries for various applications. Conductive polymers (CPs) are regarded as one of the most promising classes of functional materials capable of enhancing battery performance to address these challenges.

**Scheme 1:**
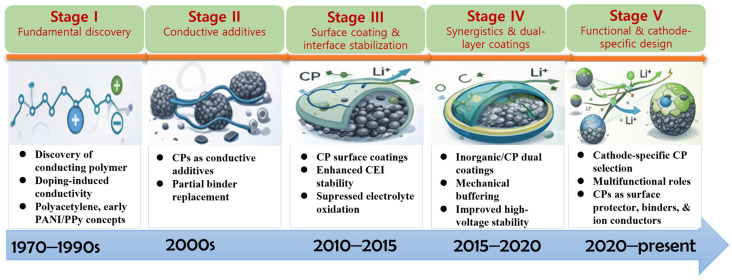
The timeline illustrates the evolution of conductive polymers (CPs) in lithium-ion battery cathode applications.

Conductive polymers (CPs) have evolved from fundamental electronic materials into multifunctional components for lithium-ion battery (LIB) cathodes. CPs such as polyaniline (PANI), polypyrrole (PPy), polythiophene (PTh), poly(3,4-ethylenedioxythiophene) (PEDOT), polyacetylene, and polysulfur nitride (PSN) and their derivatives are popular in LIBs research. Due to their inherent electronic conductivity and mechanical flexibility, CPs facilitate efficient electron transport within the electrode matrix, thereby reducing internal resistance and enhancing rate capability [24]. Following the discovery of intrinsic conductivity in π-conjugated polymers in the late 1970s, early studies in the 1990s and 2000s explored polymers such as polyaniline (PANI) and polypyrrole (PPy) primarily as conductive additives or partial binder replacements, aiming to improve electronic percolation in composite electrodes [25,26]. At this stage, CPs played a largely auxiliary role, and their application in cathodes was limited by processing challenges and insufficient electrochemical stability. A paradigm shift occurred in the 2010s, when CPs began to be deliberately engineered as surface coatings and interfacial modifiers rather than passive conductive fillers, as shown in Scheme 1. Thin CP layers were shown to simultaneously enhance electronic conductivity and stabilize the cathode–electrolyte interface, suppressing electrolyte decomposition and transition-metal dissolution in layered oxides [27]. As cathode chemistry progressed toward Ni-rich layered materials, CPs were increasingly integrated into synergistic architectures, particularly dual-layer coatings that combined chemically robust inorganic layers with conductive polymer shells to compensate for electronic insulation and mechanical brittleness [28]. More recently, CP design has become cathode-specific and function-driven, with tailored polymers addressing the dominant degradation mechanisms of layered, olivine, and spinel cathodes [29,30]. This evolution underscores a broader transition of CPs from simple conductivity enhancers to active interfacial and mechanical regulators, enabling targeted cathode design for high-energy and long-life LIBs.

When used as conductive agents, CPs also serve as flexible matrices and can buffer the volume changes in active materials during cycling. It can adapt to the mechanical stresses caused by volume expansion and contraction during charge–discharge cycles, thus reducing mechanical degradation and enhancing structural integrity [31]. Moreover, CPs can act as stabilizers of the electrode–electrolyte interface. For instance, CP layers can suppress the continuous decomposition of the electrolyte by forming a more stable and uniform solid electrolyte interphase (SEI), which reduces irreversible capacity loss and prolongs battery life [32]. Their ability to form conformal layers also helps maintain intimate contact between the electrode and electrolyte, mitigating interfacial resistance and promoting long-term cycling stability. On the cathode side, CP coatings help prevent metal dissolution and electrolyte oxidation, which are common degradation pathways in transition metal oxide cathodes [33]. When CPs have been successfully integrated into composite electrodes, they form interconnected conductive highways that enhance both electron and ion transport pathways. The porous structure of these materials provides a high surface area for the diffusion of ions and the dispersion of active materials. Their compatibility with various nanostructured materials also enables the design of high-performance electrodes with optimized porosity and electrochemical accessibility [34]. Additionally, some conductive polymers (CPs) exhibit pseudocapacitive behavior, which allows them to participate in charge storage processes, thereby contributing additional capacity through faradaic redox reactions and increasing the overall energy density of the battery [35]. It is noteworthy that the electrochemical properties and electrical conductivity of CPs can be enhanced further by doping. Doping in conducting polymers (CPs) involves the addition of chemical substances that introduce positive or negative charges into the polymer matrix [36]. This process increases the density of charge carriers, such as holes or electrons, which enhances the material’s overall conductivity. Typically, dopants are small molecules or ions that either donate or accept electrons, thereby altering the electronic structure of the polymer and improving intrinsic conductivity even further. Due to these diverse benefits, conductive polymers are being increasingly utilized in the development of next-generation LIBs to achieve greater energy density, extended cycle life, and enhanced safety.

This review article explores the application of conducting polymers in advanced LIB cathodes, detailing their synthesis methods, properties, and applications. It highlights key characteristics such as electrical conductivity, doping, thermal stability, and structural flexibility. While previous reviews have primarily addressed the use of conductive polymers in anode materials such as Si/SiO [37,38], and TiO_2_ [39], with some focus on binders [40], there remains a gap in the literature on conducting polymer–cathode hybrid composite electrodes for LIB applications. The goal of this review is to provide insights into hybrid composite cathodes, particularly in the context of advancements in rechargeable LIBs over the past decades. It examines the types of CP used to make hybrid composite electrodes and factors influencing their electrochemical properties, along with highlighting significant recent developments in this field.

## 2. Benchmark Conductive Polymers for LIBs

**Figure 1:**
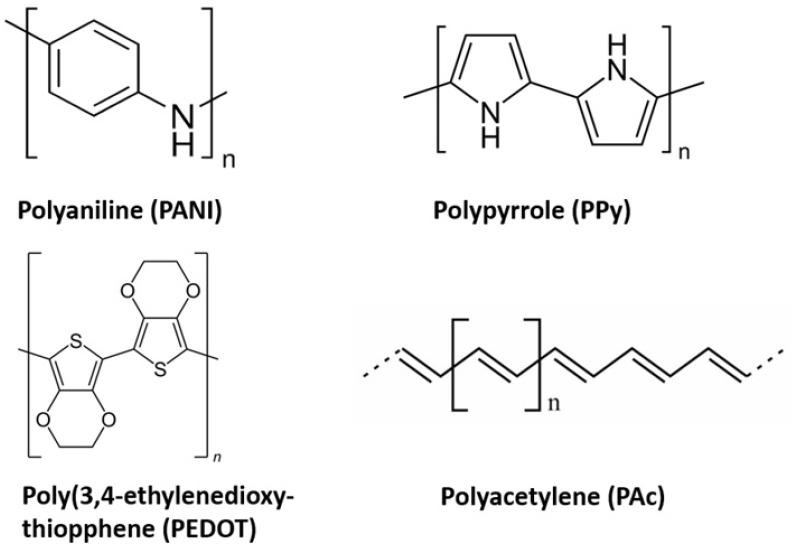
Different representative conductive polymers for battery electrode applications.

Conductive polymers can transition between the conducting and semiconducting states, making them versatile materials [41]. Chemists explain that pristine conductive polymers share a common electronic characteristic due to their structure, which consists of conjugated single and double bonds, as presented in Figure 1. Both types of bonds contain a localized sigma bond, resulting in a robust chemical bond. Generally, conductive polymers are composed of highly π-conjugated polymeric chains [42]. This means that their electronic structure allows for the delocalization of electrons across the polymer backbone, facilitating electrical conductivity. While their conductivity is typically lower than that of metals, they exhibit significantly greater conductivity than conventional insulating polymers and ceramics, making them valuable conductive components in electronic devices [43]. These materials often have the advantages of being lightweight, flexible, and less prone to corrosion compared to metals. Furthermore, they can be produced at a lower cost and with less environmental impact than traditional metal conductors [41]. Additionally, their chemical structure can be easily modified to tailor their electrical properties for specific applications, making them versatile for various electronic innovations. The highest levels of conductivity are generally attained through chemical or electrochemical doping, achieving values comparable to those of certain metals. Doping is a reversible process that introduces positive (p-doping) or negative (n-doping) charges to the conductive polymer backbone [36]. Moreover, conductive polymers can be purposefully designed and fabricated into nanostructures, offering new capabilities such as adjustable conductivity, flexibility, and a mixed conduction mechanism that reduces interfacial impedance between electrodes and electrolytes [44]. As a result, various factors, including chemical composition, molecular structure, and processing conditions, have an impact on the performance of conductive polymers. Overall, their π-conjugated backbones and appropriate doping processes make them ideal for battery electrode applications.

### 2.1. Polyaniline (PANI)

Polyaniline (PANI) exists in multiple oxidation states—leucoemeraldine (fully reduced), emeraldine (partially oxidized), and pernigraniline (fully oxidized)—each exhibiting distinct electrical properties [45]. PANI was traditionally synthesized by chemically oxidizing aniline monomers with oxidants such as ammonium persulfate or potassium dichromate under acidic conditions. However, as research on PANI advanced, alternative methods such as vapor-phase polymerization, photochemically initiated polymerization, and enzymatic polymerization were introduced [46]. These methods offer advantages such as improved control over the polymerization process, higher purity of the final product, and reduced environmental impact compared to traditional methods. Additionally, modern techniques allow the synthesis of PANI with tailored properties for specific applications. Rout et al. recently summarized the commonly used oxidants and optimal reaction parameters [41]. Morphological control over PANI can be achieved by varying the synthesis method; for example, electrospinning yields one-dimensional PANI fibers, while template-assisted approaches enable the formation of three-dimensional structures [41,47]. Electrochemical polymerization is another effective route for PANI synthesis [48]. Furthermore, PANI modified with hydrochloric acid (HCl) has been shown to exhibit significantly enhanced electrical conductivity [49].

### 2.2. Polypyrrole (PPy)

Polypyrrole (PPy) is one of the most extensively studied conductive polymers, both theoretically and experimentally. It consists of conjugated structures with alternating C–C and C=C bonds and is synthesized through the oxidative polymerization of pyrrole monomers [50]. However, the synthesis of polypyrrole can present several challenges. Controlling the molecular weight and achieving a uniform polymerization process can be difficult, leading to variations in electrical properties. Additionally, the polymerization conditions, such as temperature and oxidant concentration, need to be carefully managed to prevent unwanted side reactions and ensure high-quality material [51].

### 2.3. Polyacetylene (PAc)

Polyacetylene (PAc) was first shown to change from an insulator to a conductive polymer through specific oxidation or reduction reactions [52]. By modifying PAc, multifunctional properties, including enhanced electrical conductivity, can be achieved by tuning its decorating groups. For example, doping PAc with iodine has been shown to significantly enhance its electrical conductivity, making it comparable to metals [53].

### 2.4. Poly(3,4-ethylenedioxythiophene) (PEDOT)

Poly(3,4-ethylenedioxythiophene) (PEDOT) is a π-conjugated conductive polymer widely used in energy storage applications due to its excellent electrical conductivity, thermal stability, and processability. PEDOT consists of a repeating unit of 3,4-ethylenedioxythiophene (EDOT) monomers. The polymer backbone features delocalized π-electrons extending along the conjugated thiophene rings, enabling the formation of extended π-π stacking and intermolecular charge transport paths. PEDOT is intrinsically a semiconducting polymer, but it becomes electrically conductive when oxidatively doped (p-doped). The typical dopant used is polystyrene sulfonate (PSS), forming a water-dispersible PEDOT: PSS complex. PSS acts as a charge-balancing counterion and stabilizes the positively charged PEDOT chains [54,55].

## 3. Conductive Polymer-LIBs Cathode Composites

**Figure 2:**
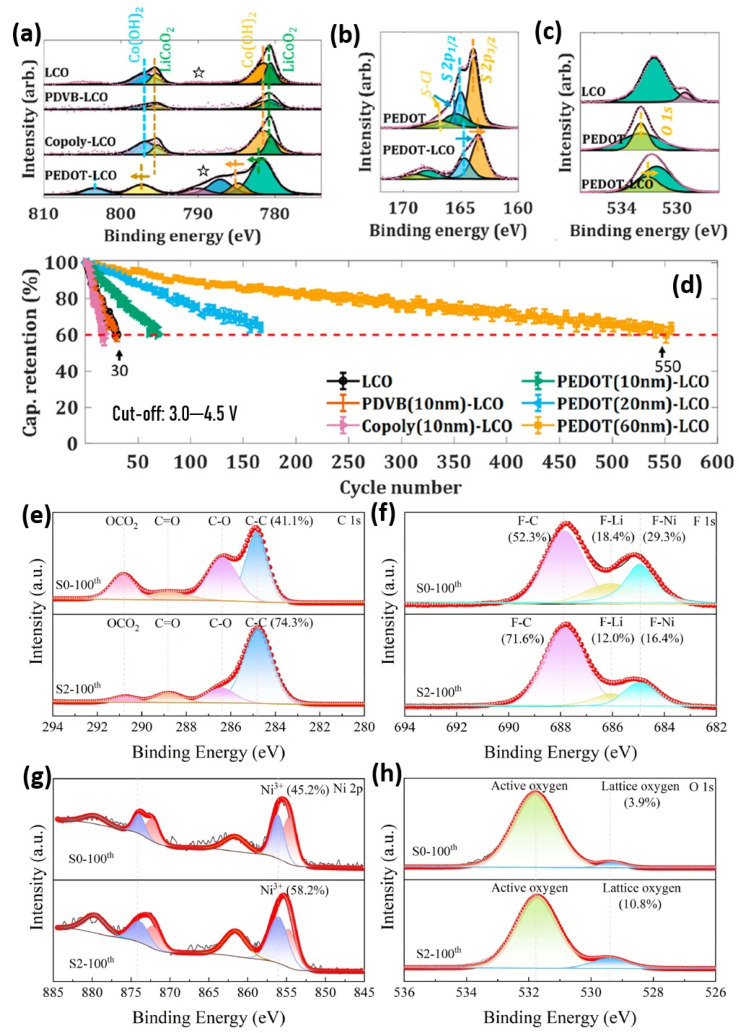
XPS data for the (**a**) Co 2p, (**b**) S 2p, (**c**) O 1s, and (**d**) electrochemical capacity retention (at 0.5 C) of bare and different polymer-coated LiCoO_2_ cathode samples. Reproduced with permission [58], Copyright ©2021 American Chemical Society. Post-cycling XPS spectra of bare (S0) and PPy-coated LiNi_0.95_Mn_0.05_O_2_ (S2) cycled at 1 C in the voltage range of 2.7 to 4.5 V (vs. Li/Li^+^): (**e**) C 1 s, (**f**) F 1 s, (**g**) Ni 2p, and (**h**) O 1 s. Adapted with permission [59], Elsevier.

Conventional cathodes, such as LiFePO_4_, LiMn_2_O_4_, and layered transition metal oxides (e.g., NCM), often suffer from limitations such as poor electronic conductivity and structural degradation during prolonged cycling [56,57]. Integrating conductive polymers (CPs) such as polyaniline (PANI), polypyrrole (PPy), and PEDOT with these cathodes helps form a conductive network that facilitates faster electron transport and reduces polarization, thereby improving rate capability and power performance. As surface modifiers, they protect cathodes from electrolyte degradation and transition metal dissolution, extending cycle life. Their mechanical flexibility further buffers the volume changes in cathode particles during repeated lithiation and delithiation, maintaining electrode integrity. Multiple studies have provided direct and complementary evidence that conductive polymer (CP) coatings suppress lattice distortion and transition-metal dissolution from cathode materials by stabilizing both surface chemistry and bulk crystal structure during electrochemical cycling. The ICP-MS measurement showed that the PEDOT coating reduced Co dissolution from 0.27% to 0.08% in LiCoO_2_ after 40 cycles [58]. In Figure 2a, XPS analysis reveals that PEDOT uniquely alters the surface electronic structure of LiCoO_2_, as evidenced by a positive shift in Co 2p binding energies compared to pristine and CP-coated samples. Similarly, reduced S 2p (Figure 2b) and O 1s (Figure 2c) binding energies of PEDOT at the LiCoO_2_ interface, indicating strong Co–S/O chemical bonding. These interfacial bonds effectively suppress cobalt dissolution and mitigate electrolyte decomposition, thereby enhancing cycling stability (Figure 2d). A post-cycling XPS analysis reveals that the PPy-coated LiNi_0.95_Mn_0.05_O_2_ (LNM95) cathode exhibits significantly reduced electrolyte decomposition compared to uncoated LNM95 (S0), evidenced by a higher proportion of C–C species and lower contents of carbonate decomposition products (Figure 2e). In addition, the PPy-coated LNM95 cathode (S2) experiences significantly less Ni and Li surface accumulation compared to the uncoated S0 sample. The reduced F–Li (12.0% in S2 vs. 18.4% in S0) and F–Ni (16.4% in S2 vs. 29.3% in S0) signals (Figure 2f) suggest that the flexible PPy layer effectively suppresses Li salt decomposition and Ni metal dissolution/migration, likely by stabilizing the cathode–electrolyte interface [59]. The higher Ni^3+^ content in S2 (58.2% vs. 45.2% in S0) indicates less Ni reduction and associated oxygen loss (Figure 2g). The significantly higher lattice oxygen retention in S2 (10.8% vs. 3.9%) suggests that the polymer coating mitigates oxygen activation and lattice distortion, thereby enhancing structural stability during cycling (Figure 2h).

**Figure 3:**
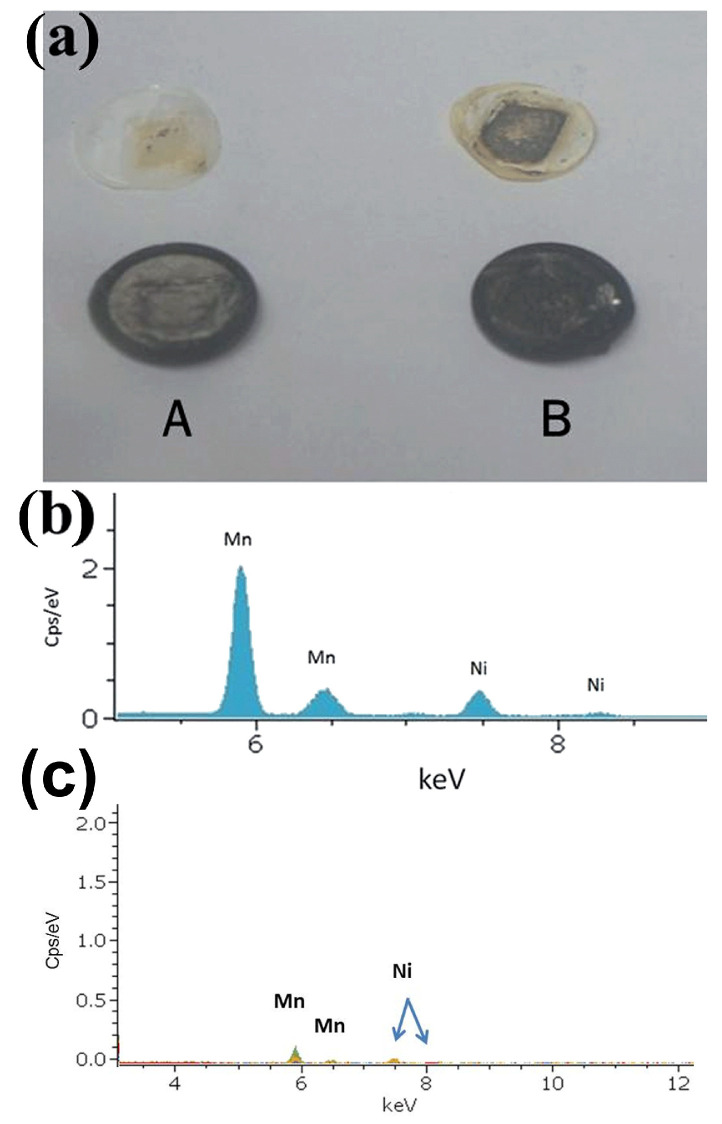
(**a**) Photographs of the lithium anodes tested against LNMO–PPy (A) and bare LNMO (B) cathodes, along with their corresponding separators. EDS spectrum of lithium anodes for (**b**) bare LNMO and (**c**) LNMO–PPy samples after 100 cycles at 55 °C. Reproduced with permission [60], Royal Society of Chemistry.

In another study, polypyrrole (PPy)-coated LiNi_0.5_Mn_1.5_O_4_ (LNMO) composites demonstrate excellent electrochemical performance, particularly at elevated temperatures (retained ~91% of its initial capacity after 100 cycles at 55 °C), significantly outperforming the uncoated LNMO [60]. Post-mortem analysis revealed severe black deposits on the Li anode and separator in the bare LNMO cell, whereas the separator in the LNMO–PPy cell remained visually clean (Figure 3a). Energy dispersive spectroscopy (EDS) analysis of the Li anode from the uncoated cell (Figure 3b) showed intense Mn and Ni signals, indicating substantial dissolution of Mn^3+^/Mn^4+^ and Ni^2+^ from LNMO and their migration to the lithium surface. In contrast, the Li anode from the LNMO–PPy cell exhibited negligible Mn and Ni peaks in the EDS spectrum (Figure 3c), confirming that the PPy coating effectively suppresses transition metal dissolution.

Additionally, a number of CPs are widely used as a binder in LIB electrode formulations, enhancing the overall performance and stability of the electrode compared to conventional PVdF or PAA binders. Herein, the progress achieved by incorporating conducting polymers into LIB electrode materials is highlighted.

### 3.1. Conductive Polymer in Layered Cathode

**Scheme 2:**
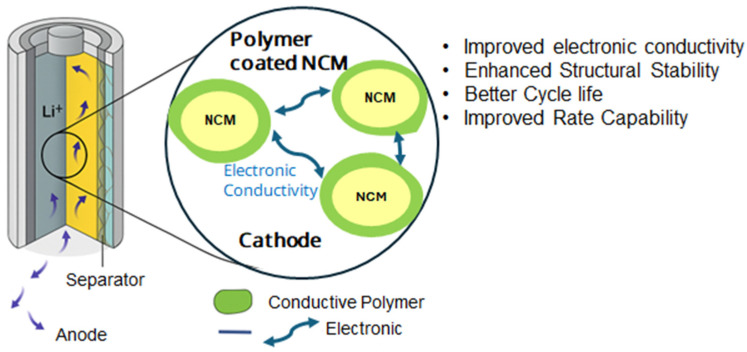
Schematic representation of a polymer-coated NCM cathode in Li-ion batteries.

Layered transition metal oxides, such as LiCoO_2_, LiNi_1-x-y_Co_x_Mn_y_O_2_ (NCM), and LiNi_1-x-y_Co_x_Al_y_O_2_ (NCA), are among the most widely used cathode materials in commercial LIBs due to their high energy density and well-defined layered crystal structure. Their major drawbacks are cation-mixing, limited electronic conductivity, electrolyte degradation, transition metal dissolution, and structural instability at high voltages or prolonged cycling [61,62]. Conductive polymers such as polyaniline (PANI), polypyrrole (PPy), and poly(3,4-ethylenedioxythiophene) (PEDOT) possess intrinsic electronic conductivity and can form percolating networks or coatings on these cathode particles. These CP coatings and networks facilitate electron transport within the electrode, thereby improving rate capability and reducing polarization losses (Scheme 2). For example, J. Cao et al. demonstrated that LiCoO_2_ coated with PPy exhibited a significant reduction in charge-transfer resistance and enhanced capacity retention at high current densities, owing to the improved electron percolation pathways provided by the polymer layer [63].

**Figure 4:**
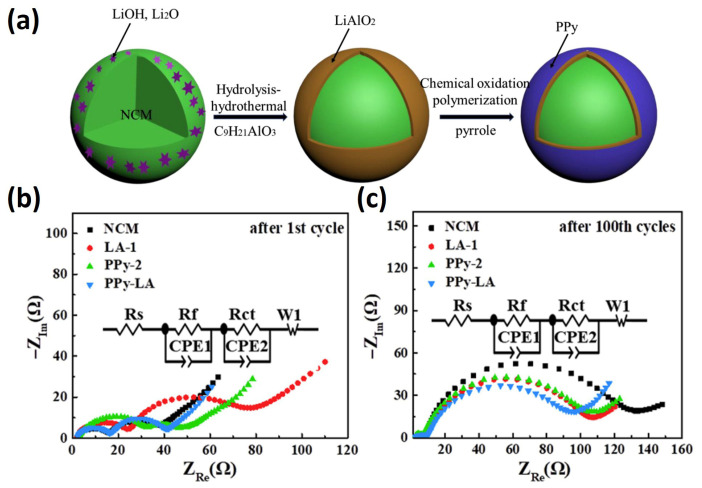
(**a**) Schematic representation of dual-conductive coating on NCM cathode using LiAlO_2_ and PPy. Nyquist plots of bare NCM, LiAlO_2_ coated NCM (LA-1), PPy coated NCM (PPy-2) and LiAlO_2_–PPy dual–coated NCM (PPy-LA) samples after (**b**) 1st cycle and (**c**) 100th cycles; reproduced with permission [65], Elsevier.

Several studies have demonstrated the effectiveness of polypyrrole (PPy) coatings in enhancing the electrochemical performance of high-nickel NCM cathodes. N. Haolong et al. [64] demonstrated that electrochemical in situ polymerization of PPy on NCM cathodes forms a uniform conductive layer that stabilizes the electrode–electrolyte interface while accelerating Li^+^ diffusion and charge transfer. The pyrrole monomer was introduced into a commercial electrolyte containing LiPF_6_, ethylene carbonate, diethyl carbonate, and ethyl methyl carbonate. Under high potential, oxidation and electro-polymerization occurred, forming a uniform conductive layer on the cathode surface, which stabilized the interface and facilitated fast Li^+^ diffusion and charge transfer. Y. Ma et al. used polypyrrole (PPy) as a dual conductive coating material with LiAlO_2_ on a high-nickel NCM cathode using a hydrolysis–hydrothermal method and in situ chemical polymerization (Figure 4a) [65]. The optimal PPy coating was determined to be 2 wt%. Using the EIS technique, they analyzed the cathode material interface reaction kinetics and electrode resistance, demonstrating that PPy facilitates rapid electron transfer, as shown in Figure 4b,c. The resistance of PPy-coated samples decreased with increasing cycles, indicating that the surface-modified samples inhibit harmful side reactions at the cathode–electrolyte interface.

**Figure 5:**
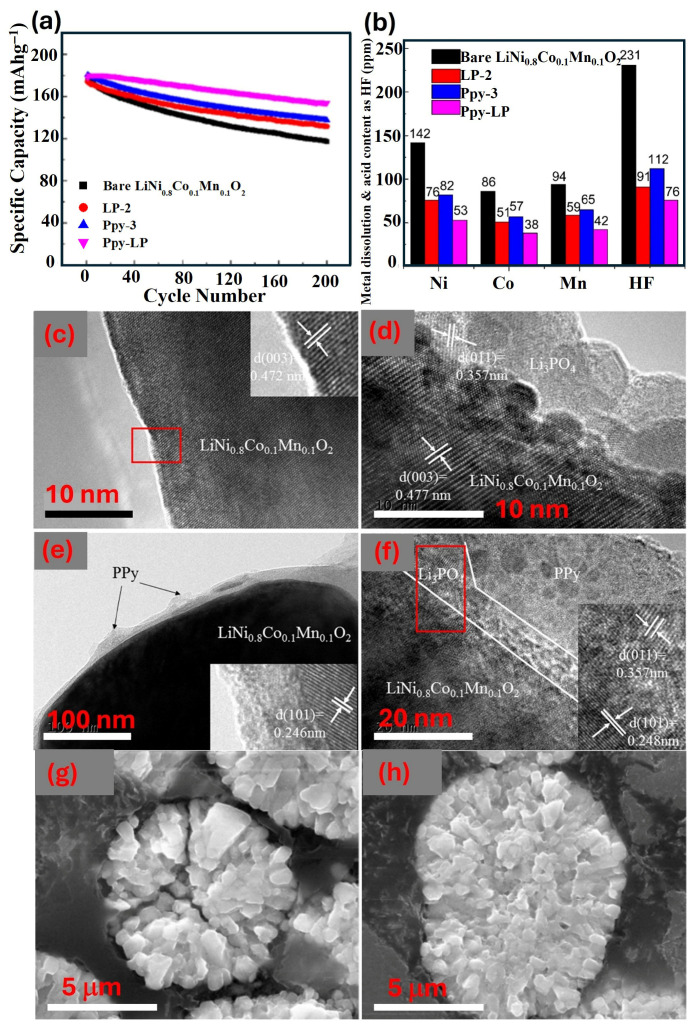
Electrochemical and morphological characteristics of bare and three surface-modified NCM811 samples. (**a**) cycling performance at 1 C, (**b**) the amount of dissolved transition metal ions (in ppm) in the electrolyte after 50 cycles according to HF titration results. TEM images of (**c**) bare NCM811 (demonstrating representative NCM lattice fringe d_003_ = 0.472 nm), (**d**) LP-2 (distinct Li_3_PO_4_ coating layer on the NCM surface), (**e**) PPy-3 (amorphous PPy coating layer on the NCM surface), and (**f**) PPy-LP samples (representing Li_3_PO_4_-PPy dual-coating layer). Cross-sectional SEM images of (**g**) bare NCM811 (×20,000 magnification), (**h**) PPy-LP (×20,000 magnification) cathode materials after 50 cycles. Reproduced with permission [66]. Copyright ©2017 American Chemical Society. Herein, LP-2: Li_3_PO_4_ coated NCM811, PPy-3: Polypyrrole coated NCM811, and PPy-LP: dual-coated (Li_3_PO_4_ and polypyrrole) NCM811 sample. The red box marks the area selected for the corresponding SAED pattern displayed in the inset.Q. Gan et al. achieved a uniform coating of conducting PANI on LiNi_0.8_Co_0.1_Mn_0.1_O_2_ (NCM811) surface using PVP as an inductive agent [71]. The uniform and thin PANI conductive layer was achieved through bonding between PVP and PANI molecules, where PVP can serve as a “bridge” to link NCM811 and limit the bonding amount of PANI (Figure 6a,b). The PANI layer was found to be uniform and thin, as depicted in HRTEM images (Figure 6c,d), resulting in improved electrochemical performance and cycling stability (Figure 6e,f). The rate capability test reveals that NCM811 materials without the PANI layer experience a significant drop in working voltage, while NCM811@PANI-PVP shows minimal drop even at high rates (Figure 6g). In addition, superior long-term cyclability was also observed at 200 mA g^−1^ for NCM811@PANI-PVP (Figure 6h). The GITT test indicates that NCM811@PANI has a higher Li-ion diffusion coefficient than the bare NCM811, which accounts for the superior rate capability of the PANI-coated cathode sample.

S. Chen et al. successfully synthesized high-nickel NCM811 composites coated with PPy-Li_3_PO_4_, showing improved cycle stability and rate performance compared to the bare material [66]. The bare NCM cathode experiences significant capacity decline (Figure 5a) and structural disintegration due to the dissolution of transition metals and HF attack (Figure 5b). Lithium residues accelerate the hydrolysis of LiPF_6_, producing more LiF, H_2_O, HF, and Li_x_POF_y_-type compounds [67,68]. The Li_3_PO_4_ coating suppresses electrolyte decomposition and the dissolution of transition metals from the active cathode materials into the liquid electrolyte, maintaining the structural stability of the active materials (Figure 5b–d). The PPy secondary coating compensates for defects in the Li_3_PO_4_ coating layer, preventing direct contact between the bulk material and electrolyte, inhibiting surface side reactions, increasing electronic conductivity between particles, and reducing internal crack formation (Figure 5e,f). Cycled Li_3_PO_4_-PPy-coated samples showed significantly fewer cracks compared to the cycled bare samples (Figure 5g,h). In another study, Li_2_SiO_3_-PPy dual-coating on the NCM811 surface resulted in an initial specific capacity of 145.1 mAh g^−1^ and a retention rate of 96.4% at 10 C, which is much higher than that of its bare counterpart (~82.2 mAh g^−1^ with 84.5% retention) [69]. The dual-conductive layers of Li_3_VO_4_ and polypyrrole (PPy) are successfully coated on the spherical LiNi_0.6_Co_0.2_Mn_0.2_O_2_ (NCM622) particle surface, preventing transition metal dissolution from the crystal structure. The dual-coated NCM sample demonstrates excellent cycling stability, achieving a retention rate of 93.7% after 100 cycles, compared to 73.6% for the pristine NCM, at 0.5 C under a high cut-off voltage of 4.5 V [70]. These performances highlight the effectiveness of the dual-coating approach in enhancing electrochemical stability and capacity retention under high-rate cycling. The inorganic coating layer provides a robust protective barrier, while the conductive polymer coating improves conductivity, synergistically contributing to the overall efficiency and durability of the NCM811 cathode material. Peng He et al. investigated the improvement mechanism of a PPy coating layer on a Co-free and Ni-rich LiNi_0.95_Mn_0.05_O_2_ (NM95) cathode surface [59]. The PPy layer provides physical protection to the cathode, hindering electrolytic erosion and reducing surface degradation. Its high flexibility allows it to adapt to volumetric changes in NM95 particles during charge/discharge, reducing micro-cracks and preventing further eroding of the fresh NM95 surface by the electrolyte. In a nutshell, in situ electrochemical polymerization of PPy on NCM creates a uniform conductive layer that enhances Li^+^ diffusion and interfacial stability. Further improved performance can be achieved with a dual LiAlO_2_–PPy coating, which optimizes interfacial kinetics at 2 wt% PPy and lowers charge-transfer resistance. A Li_3_PO_4_–PPy bilayer effectively suppresses harmful side reactions, enhances conductivity, and minimizes microcracking. Similarly, a Li_2_SiO_3_–PPy dual-coating provides high-capacity retention (96.4% at 10 C), demonstrating durability under fast-charging conditions. A Li_3_VO_4_–PPy coating on NCM622 supports long-term cycling at high voltage (4.5 V) with 93.7% retention after 100 cycles. Finally, this approach can be extended to Co-free Ni-rich NM95 cathodes, where the flexible PPy layer mitigates surface degradation and microcracking, showcasing its potential for next-generation cobalt-free LIB cathodes.

**Figure 6:**
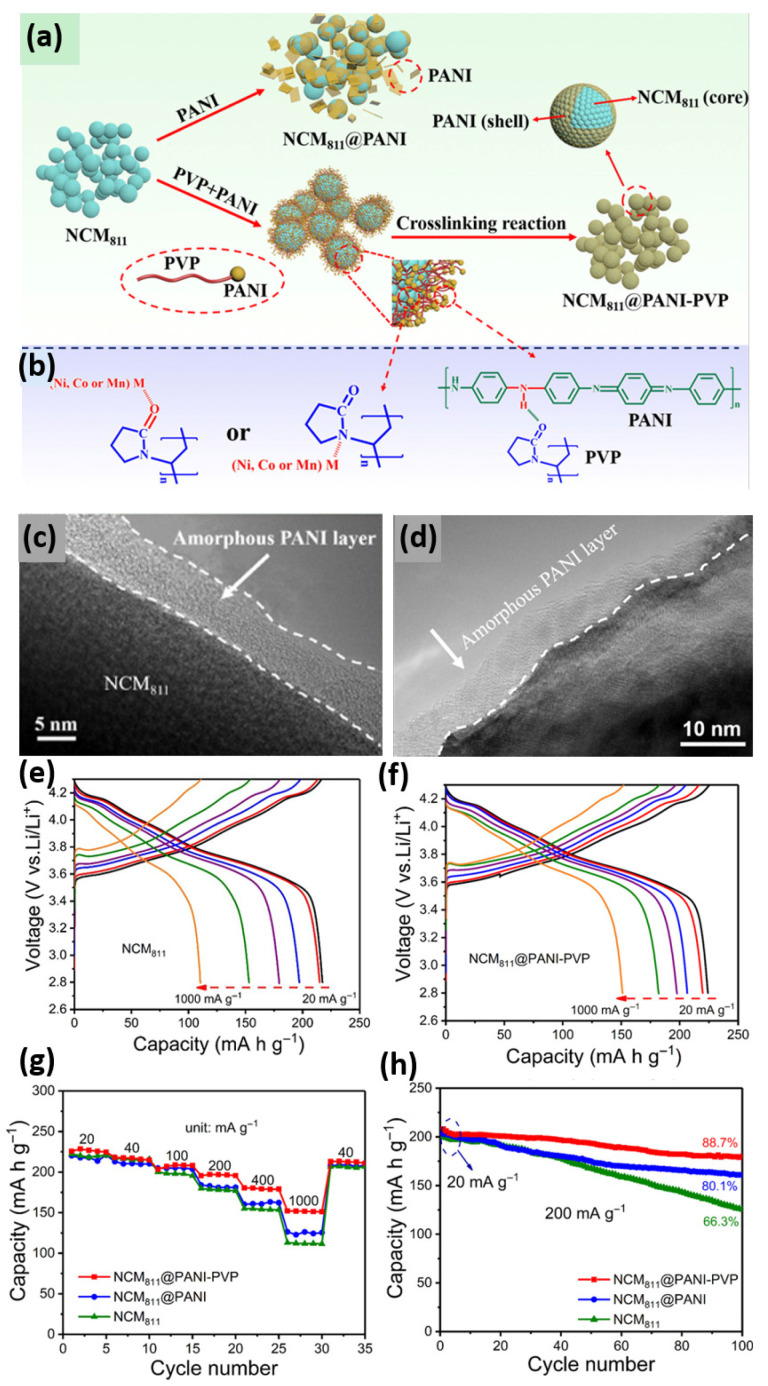
(**a**) Schematic representation of the preparation process for NCM 811 @PANI–PVP, (**b**) possible reaction among NCM 811, PVP, and PANI. (**c**,**d**) HRTEM images of NCM 811@PANI-PVP demonstrate a distinct amorphous PANI coating layer on the NCM surface. Charge–discharge voltage profiles of the (**e**) NCM 811 and (**f**) NCM 811@PANI-PVP. Comparison of the (**g**) rate performance, and (**h**) cycling performance of NCM 811, NCM 811 @PANI, and NCM 811 @PANI–PVP. Reproduced with permission [71], Copyright ©2019 American Chemical Society.

**Figure 7:**
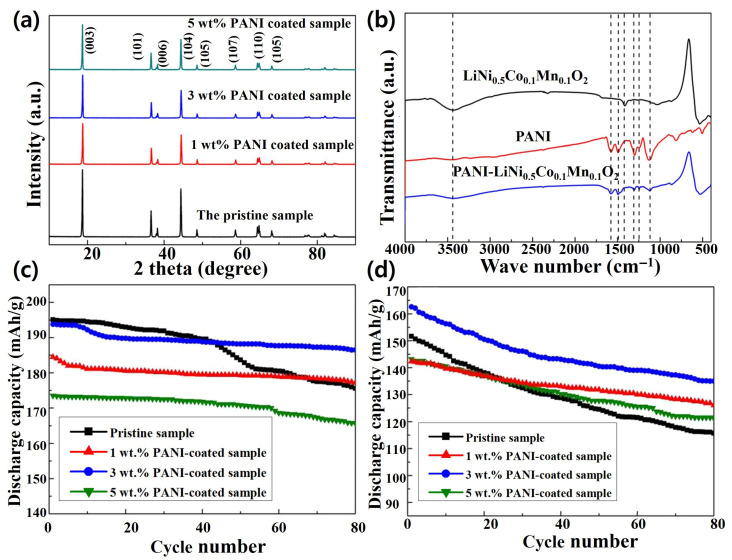
(**a**) XRD patterns, (**b**) FTIR spectrum, and electrochemical cycling performance at (**c**) 1 C, and (**d**) 5 C of pristine and PANI-coated NCM 811 samples. Reproduced with permission [72], Copyright © 2018, Springer Nature.

In another study, a polyaniline (PANI)-coated NCM811 cathode material has been developed by Z. Xiao’s group to prevent lithium discharge and structural damage [72]. In Figure 7a, the XRD peak intensity ratios of (003)/(104) for pristine and PANI-coated NCM811 samples are 1.38 (pristine), 1.39 (1 wt.%), 1.55 (3 wt.%), and 1.49 (5 wt.%). The cationic mixing between Li^+^ and Ni^2+^ is minimal when the intensity ratio of I_(003)_/I_(104)_ exceeds 1.2. This indicates that the Li^+^ ions are more likely to occupy the Li^+^ layers, while the Ni^2+^ ions remain in their designated transition metal layers. This structural arrangement enhances the stability and electrochemical performance of the material, as it minimizes disruptions in the layered framework. In FTIR analysis (Figure 7b), the characteristic peaks at 1422 cm^−1^ and 3440 cm^−1^ are associated with C=O and O–H bands, corresponding to Li_2_CO_3_ and LiOH, respectively. In the PANI-coated NCM811 material, these peaks were significantly weakened, indicating a decrease in surface residual alkali. Therefore, the PANI coating effectively reduces cationic mixing and surface residual alkali content, optimizing interfacial electrochemical reactions and enhancing cathode stability and safety. The coating samples demonstrate enhanced capacity retention: 96.25% at 1 C and 83.02% at 5 C after 80 cycles, which was far better than that of the pristine sample (Figure 7c,d).

**Figure 8:**
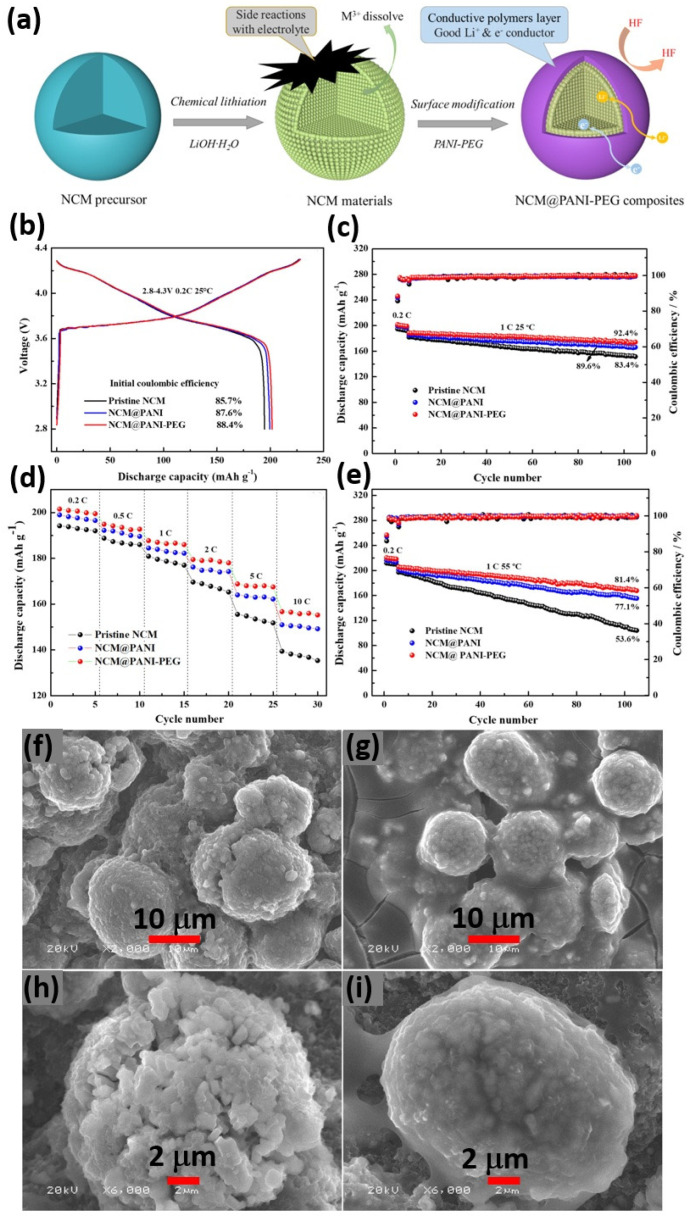
(**a**) Schematic representation of the NCM@PANI–PEG composite synthesis. Electrochemical performance of NCM, NCM@PANI, and NCM@PANI–PEG. (**b**) first charge–discharge voltage profile, (**c**) cycling test at 25 °C, (**d**) rate capability, (**e**) cycling test at 55 °C. Morphology of cycled electrodes: (**f**,**h**) NCM and (**g**,**i**) NCM@PANI–PEG. Adapted with permission [73]. Copyright ©2018, American Chemical Society.

Y. Cao et al. proposed that a conductive polyaniline–PEG hybrid coating can improve the rate performance and cycling stability of LiNi_0.8_Co_0.1_Mn_0.1_O_2_ (NCM) cathode materials (Figure 8a) [73]. The PANI–PEG polymers, with their elasticity and flexibility, play a crucial role in mitigating volume contraction or expansion of the host materials during cycling. The discharge-specific capacity of the bare NCM cathode decreased rapidly over 100 cycles, whereas the NCM@PANI and NCM@PANI–PEG cathodes retained 89.6% and 92.4% of their capacity, respectively (Figure 8b,c). The rate performance of NCM@PANI and NCM@PANI–PEG electrodes was superior to that of the bare NCM, particularly at high rates (Figure 8d). The fading rate of NCM cathode materials was faster at 55 °C compared to 25 °C (Figure 8e), due to increased transition metal dissolution caused by HF erosion and electrolyte decomposition. Surface-modified samples exhibited superior capacity retention over 100 cycles at 1 C. The dissolved transition metals increased over time, impacting the electrochemical properties. The surface-modified NCM maintained a well-defined spherical shape and a visible film on the host material (Figure 8f–i), contributing to superior electrochemical performance.

**Figure 9:**
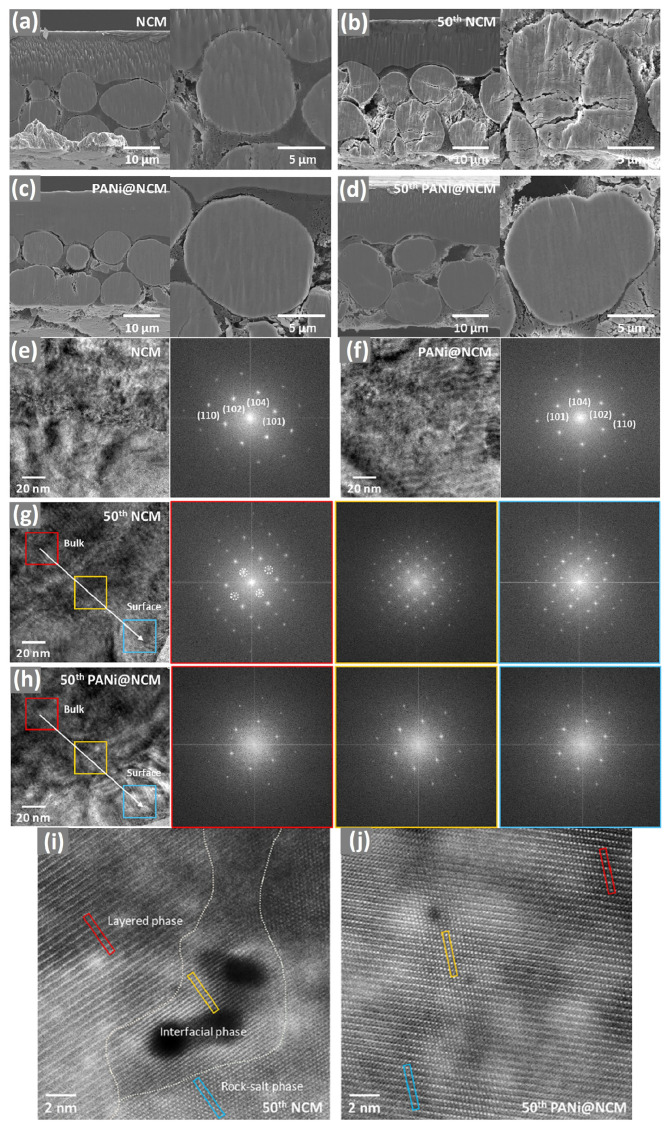
SEM images of pristine NCM in cross-sectional view (**a**) before and (**b**) after 50 cycles, and PANI@NCM (**c**) before and (**d**) after 50 cycles. TEM images and the corresponding FFT patterns of (**e**) pristine NCM, and (**f**) PANI@NCM before the cycle test; (**g**) pristine NCM, and (**h**) PANI@NCM after the cycle test. STEM-HAADF images of (**i**) pristine NCM and (**j**) PANI@NCM after the cycle test; reproduced with permission [74]. Copyright ©2023 American Chemical Society. The SAED pattern here corresponds to the red, yellow, and blue boxes, representing the bulk to surface regions of the sample.

Moreover, the mechanical flexibility of CPs offers a buffering effect that helps prevent microcracking and pulverization of cathode particles caused by repeated volumetric changes during lithiation and delithiation. This cushioning effect is particularly beneficial for high-nickel cathode systems such as NCM811, where mechanical failure is more common. The W. Shin group demonstrated that PANI-coated NCM811 cathodes exhibited improved structural integrity with cycle life over 100 cycles and avoided micro-crack, attributed to the dual roles of PANI in conductivity enhancement and mechanical reinforcement [74]. Usually, micro/macrocracks form due to continuous cathode degradation, and complicated side reactions occur on the unprotected surface of the cathode material. These cracks provide new active surfaces, making the cathode electrolyte interphase (CEI) layer thicker, which inhibits Li^+^ diffusion. The bare NCM showed widespread micro/macrocracks, and the secondary particles were split into primary particles along the grain boundaries (Figure 9a,b). In contrast, the PANI@NCM maintained smooth and crack-free particles even after 50 cycles (Figure 9c,d), with a less developed CEI layer compared to NCM without a coating layer. Moreover, the PANI coating layer on nickel-rich NCM prevents cation mixing and side reactions. High-nickel layered cathode materials often encounter irreversible phase transitions and cation mixing due to the movement of Ni^2+^ ions into Li sites, which occurs because the sizes of the ions are similar (Ni^2+^: 0.069 nm; Li^+^: 0.076 nm) during the delithiation process. HR-TEM analysis with FFT patterns and HAADF images identified phase transitions on the bare cathode surface, which is due to the cation mixing and side reactions with the electrolyte (Figure 9e,g,i). In contrast, PANI@NCM exhibited a uniform layered phase with distinct TM/Li sites in all regions (Figure 9f,h,j).

**Figure 10:**
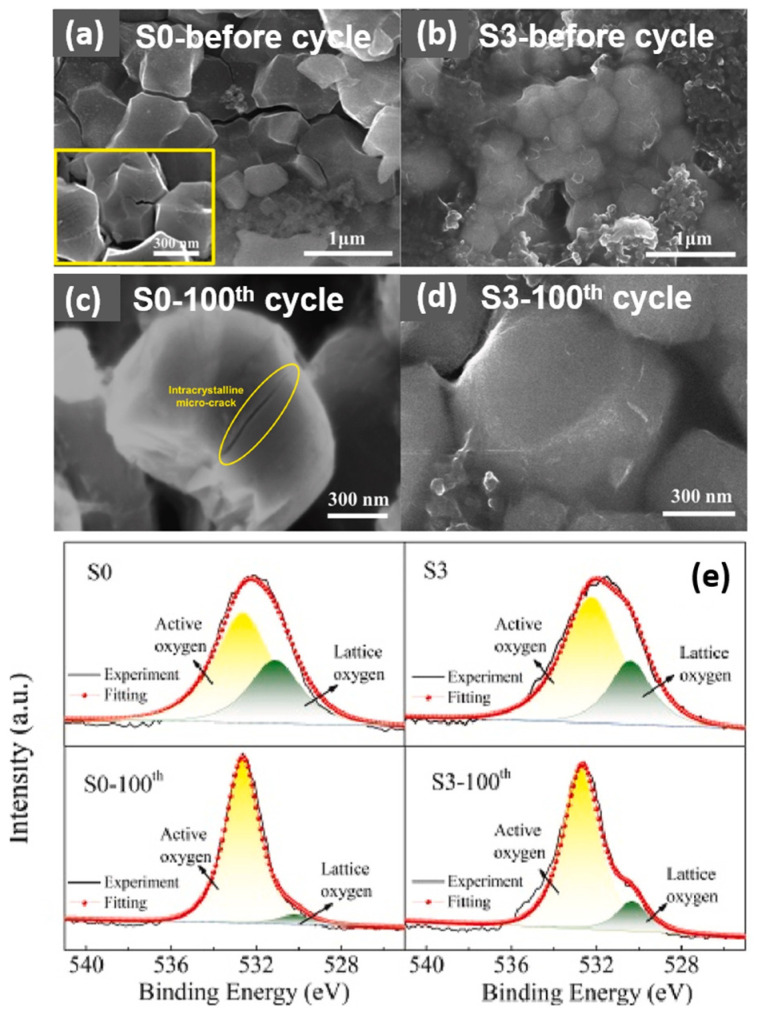
SEM images of cathode prepared by samples (**a**,**c**) S0, and (**b**,**d**) S3, (**e**) O1s XPS spectra of S0 and S3 cathodes, taken before and after 100 cycles, reproduced with permission [75], Elsevier. Herein, S0 denotes bare NCM811, and S3 denotes EPS-coated NCM811. The yellow circle in (c) indicates crack formation in the bare sample after cycling.

Cracks are categorized as intergranular or intracrystalline, and while many coating studies have effectively addressed intergranular cracks, intracrystalline cracks in primary particles, such as those found in the field, sometimes remain under-researched. X. Tan et al. [75] applied ethoxy functional polysiloxane (EPS) coating on an NCM811 cathode material via tetraethyl orthosilicate (TEOS) hydrolytic polycondensation without additives to address intracrystalline micro-cracks in primary-like particles (Figure 10a–d). EPS-coated NCM811 achieved a high-capacity retention rate of 95.6% at 1 C, attributed to the EPS stress buffer layer, reduction of oxygen vacancies, inhibition of phase transitions, and minimized volume changes (Figure 10e). Similarly, another study found that polysiloxane coating enhances the electrochemical properties of Ni-rich NCM811 material [76]. Tetraethyl orthosilicate (TEOS) is hydrolyzed to form a polysiloxane coating on the NCM811 surface through hydrolysis with trace water present on the cathode surface. This coating protects the cathode by removing residual H_2_O from the cathode surface and preventing the reaction between HF in the electrolyte and NCM, thereby inhibiting interfacial side reactions. The polysiloxane layer also blocks the electrolyte from penetrating the cathode particles, which prevents the formation of microcracks and improves cycling performance.

**Figure 11:**
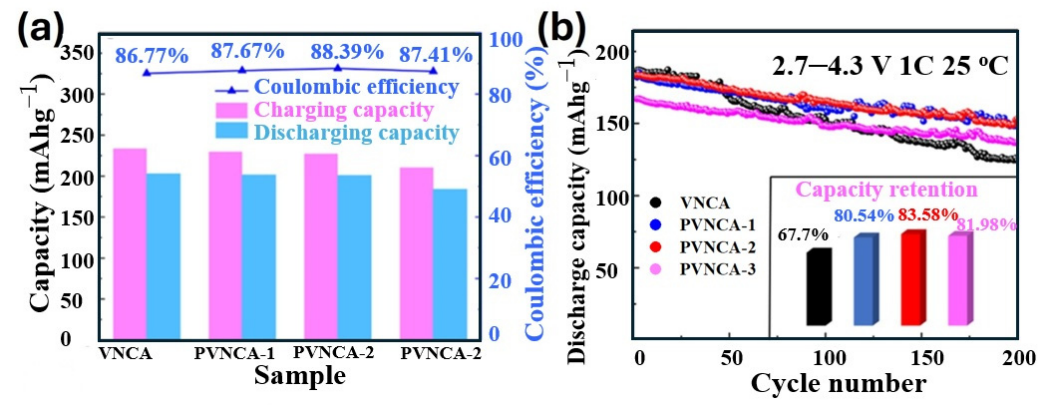
Comparison of (**a**) initial specific charge–discharge capacity and coulombic efficiency, and (**b**) cycling performance (at 1 C) of bare VNCA, PEDOT: PSS coated PVNCA-1 (1 wt.%), PVNCA-2 (2 wt.%), and PVNCA-3. (3 wt.%) electrode samples; reproduced with permission [77], Elsevier.

In addition to enhancing conductivity, CPs function as interfacial stabilizers. When used as surface coatings, they mitigate adverse reactions between the cathode and liquid electrolyte, especially at high voltages where electrolyte oxidation and transition metal dissolution are prominent. For instance, PEDOT: PSS has been applied as a surface modifier for V-doped NCA cathodes, successfully reducing interfacial impedance and improving capacity retention during cycling (Figure 11) [77]. The ionically permeable yet electronically conductive nature of these polymers allows for efficient lithium-ion diffusion while maintaining a stable electrode–electrolyte interface. This stabilization effect is crucial for improving the long-term performance and safety of lithium-ion batteries. By reducing interfacial impedance, CPs enhance charge transfer efficiency, while their protective role minimizes the risk of thermal runaway and capacity fading, making them indispensable in advanced battery designs. Z. Jia et al. demonstrated that a low-cost polyethylene (PE) coating on the surface of Ni-rich materials (NCM811) significantly reduces the side reactions at the electrolyte-material interface, improves the structural stability of the materials during cycling, and lowers the charge transfer resistance [78]. Kim et al. [79] applied polyimide/polyvinylpyrrolidone (PI/PVP) for hybrid coating on a Co-free hi-nickel cathode, LiNi_0.96_Mg_0.02_Ti_0.02_O_2_ (NMT), to suppress side reactions, resulting in a capacity retention of 86.7% at C/3 rate after 500 cycles.

Polyacrylonitrile (PAN) was transformed into a cyclized polyacrylonitrile (cPAN) layer through heat treatment and calcination, which is anticipated to improve electronic conductivity. The cPAN-coated NCM622 showed enhanced electrochemical performance, with the thickness of the cPAN significantly influencing high-rate performance [80]. Specifically, a 2% PAN coating led to superior performance compared to other thicknesses. Electrochemical impedance spectroscopy (EIS) after 30 charge/discharge cycles revealed that the cPAN–NCM samples exhibited significantly lower charge transfer resistance (R_ct_) than bare NCM, which had an increased R_ct_ due to an unstable cathode–electrolyte interphase (CEI) formed from electrolyte decomposition at high voltages. Additionally, the presence of cPAN was effective in preventing crack formation in cathode particles over an extended cycling period of 100 cycles. A new polymer coating based on lithium sulfonyl(trifluoromethane sulfonyl)imide methacrylate (LiMTFSI) is applied to NCM811 cathodes, improving electrochemical stability at high C-rates and voltages up to 4.5 V, compared to uncoated cathodes [81]. This coating reduces charging times, preserves cycling performance, prevents surface phase transitions to the rock-salt phase, minimizes transition metal dissolution, and enhances lithium-ion transport at the cathode–electrolyte interface.

**Figure 12:**
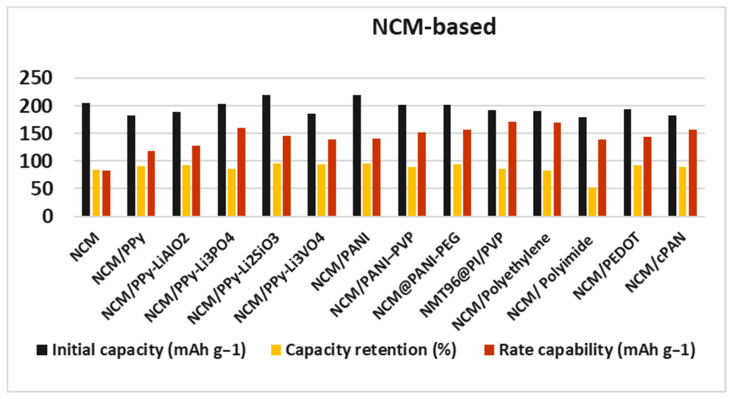
Comparison of the initial capacity, capacity retention, and rate capability of various developed NCM-based polymer composite cathodes.

In addition to their structural and interfacial roles, certain CPs also provide pseudocapacitive charge storage, contributing extra capacity through reversible redox reactions. While their contribution to the total capacity is relatively small compared to layered oxides, it can be advantageous in high-rate or thin-film configurations where surface reactions are predominant [24,82,83,84]. The versatility of CPs is further highlighted by their ability to be processed into various nanostructures and their compatibility with scalable fabrication techniques, such as in situ chemical polymerization, electrochemical deposition, and solution casting [85]. For example, Xiao et al. used in situ oxidative polymerization to apply a uniform PEDOT layer onto NCM particles, resulting in composite cathodes with improved thermal stability and superior rate performance [86]. A comparison of the electrochemical performances of layered electrode materials with and without polymer coatings is presented in Table 1 and Figure 12 (bar graph). The comparison reveals significant differences in performance metrics, such as charge storage capacity, capacity retention, and rate capability.

### 3.2. Conductive Polymer in Olivine-Type Cathode

Olivine structure-based polyanionic cathode materials are recognized as a significant category of positive electrode materials in the field of lithium-ion batteries. Compared to layered oxide cathodes, olivine structure-based cathodes offer superior thermal stability and safety [89,90,91]. While they may have a lower energy density than some other cathode types, their stability makes them well-suited to applications where safety is a priority. In this group, LiFePO_4_ (LFP) is considered a major contender due to prolonged cycle life and eco-friendliness in comparison to layered-based oxides such as Ni and Li–rich layered oxides [92,93]. While LFP cathodes have advantages over layer-based materials, they are inherently limited by slow Li diffusion and restricted electron transfer kinetics at high current densities and low operating temperatures below −10 °C. Strategic engineering of optimization is carried out by reducing the particle size and coating with carbon to shorten the diffusion path of the Li within the particles and promote the electron transfer within the cathode with reduced polarization. However, an increase in the surface area of nanosized particles enhances the interface electrochemistry that causes the deterioration of the structure [94,95]. Nano-sized materials show a higher moisture capability than micro-sized LFP, which causes the hydrolysis of the LiPF_6_ and produces the corrosive HF, resulting in capacity fading by promoting the iron dissolution and deposition of iron on the anode [96].

Various strategic approaches were adopted to protect the LFP iron dissolution in the electrolyte. Delaporte et al. coated the LFP with carbon by chemically grafting a trifluoromethylphenyl group to increase the hydrophobicity, consequently enhancing the stability in deionized water and reducing the iron dissolution in the electrolyte [97]. The Guyomard group utilized the amine silano group (N–Si), which has affinity towards the Fe to barrier the dissolution from LFP and directly capture the corrosive HF in the electrolyte [98]. Additionally, to protect the LFP, different electrolyte additives are utilized to create the cathode electrolyte interface (CEI). Vinylene carbonate, N-dimethyl formamide, and ethoxy (pentafluoro) cyclotriphosphazene are utilized to increase the stability of the lifespan of LFP/Li half-cells by forming CEI layers. But such approaches cause the sluggish kinetics of electron transfer in LFP cathodes, though the development suppresses the iron dissolution from the cathode [99,100,101,102].

**Figure 13:**
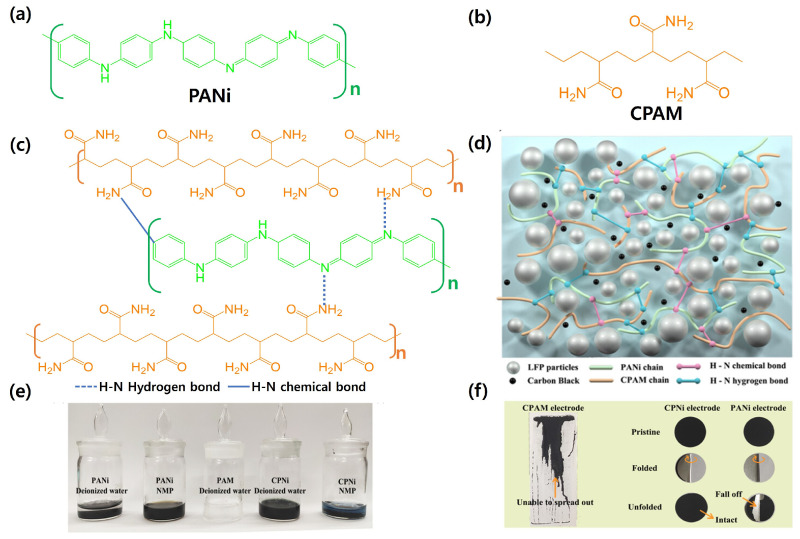
Illustration of (**a**) PANi, (**b**) CPAM, and (**c**) CPNi. (**d**) Schematic of the LFP electrode with the CPNi. (**e**) Dissolution effects of various polynemes on the DI water and NMP. (**f**) Properties of different binders on the current collector. Reproduced with permission [103], copyright 2025, John Wiley and Sons.

An alternative approach involves using conducting polymers as binders to improve performance. Combining the conducting polymers with binders may suffer from a low adhesion nature or mechanical stringent properties during the charge–discharge process. Weng group planned to prepare incorporating the conducting polymers of polyaniline (PANI) on flexible binders of cationic polyacrylamide (CPAM) to maintain the stretchable network that helps to retain the stable structural morphology during the charge–discharge process [103]. The amide group of polyacrylamide and amidogen (–NH_2_) from the PANi formed the N–H hydrogen bond and polymerization forms a stretchable network that retard the structure of the electrode from collapsing (Figure 13). Their study highlights that the dual-crosslinking network of CPNi, formed by chemical bonds and intermolecular hydrogen bonds, is expected to be stronger than a noncovalent binder, while PANI enhances cycling performance. Improved electrical conductivity from the PANI helps to reduce the electrochemical polarization due to a good conducting network, which, through the conjugated network, provides strong interactions between the LFP particles and current collectors, improves the rate capability, and cycling performances. CPNi6 shows the discharge capacity of 120 mAh g^−1^ at the 200th cycle at 1 C current density and 118 mAh g^−1^ capacity at 5 C current density [103].

Shao et al. utilized the polyethyleneimine (PEI) functionalized by the N–ethyl carboxylic acid as a functional conductive polymeric binder in considering the ion conductivity and interfacial adhesion properties [104]. The study has synthesized the N–carboxyethyl polyethyleneimine (N–CEPEI) by functionalizing the PEI with a carboxylic functional group utilizing the acrylic acid through the Michael addition reaction. Carboxylic acid optimization is usually utilized for tuning the interfacial adhesion nature between the surfaces of materials and films [105,106,107]. In here, abundant carboxylic groups help to establish a robust 3D crosslinked polymer network by in situ condensation reaction, resulting in enhanced adhesion nature and mechanical stability, and maintain structural integrity. The LFP electrode with N–CEPEI conductive polymer shows a capacity of 139 mAh g−1 with a capacity retention of 94.8/% after 400 cycles at 1 C in comparison to PVDF–LFP 86.6% capacity retention. Also, at the high rate (5 C) operation, N–CEPEI–LFP maintains the 80 mAh g^−1^ capacity after 500 cycles. LFP electrode with the carboxylic functionalized PEI provides the improved electronic and ionic conductivity results of the uniform dispersion of materials, which helps to efficient Li–ion diffusions as well as a 3D crosslinked porous structure, efficiently facilitates the electron transport and Li–ion diffusions with a robust mechanical structure to maintain the cycling stability [96]. A recent study by Yang et al. designed zwitterionic polymers (ZIPs) as a binder where the ethylene oxide units on the zwitterionic segments show efficient Li ion transportation, as it is usually used as a polymer electrolyte. It provides the lithium-ion diffusion coefficient of 3.48 × 10^−12^ cm^2^ s^−1^ [108]. As zwitterions, electrolytes with charged polar moieties are helpful for Li ion diffusion, and polar moieties increase the adhesion to the cathode materials. The study designed the ZIP of copolymerization of poly (ethylene glycol) methyl ether methacrylate (PEGMEA) and sulfobetaine methacrylate (SBMA) to maintain good adhesion and maintain ZIPs insoluble in liquid electrolytes, which facilitates the maintenance of structural integrity. The ZIP/LFP cathode shows the capacity of 111 mAh g^−1^ at 500 cycles at 3 C with 93.4% capacity retention, which is 1.1 times higher in comparison to LFP/Li half-cells [108].

Conductive polymer polypyrrole (PPy) is utilized as a host material for Li–ion inser-tion/extraction in the range of 2–4.5 V vs. Li/Li^+^ and shows the theoretical capacity of 72 mAh g^−1^. The composition of PPy with LFP enhances capacity performance by ~20% and protects the LFP material, yielding ~99% capacity retention relative to 88.8% for un-modified LFP after 30 cycles. It is a strategic approach to combine PPy with the LFP to develop performance and LFP protection purposes. To fully utilize the theoretical capacity of the LFP, the active particles of the LFP are coated with carbon [109]. Carbon coating on the LFP allows the Li–ion permeation and provides electrons that move with the Li atoms into/out of the oxide. Goodenough group systematically incorporates conductive polymer of PPy or polyaniline (PANI) with the carbon-coated LFP (C–LFP) and compares the performance of the cathode materials [110]. The study shows that electrochemically active conductive polymers overlap the energy of the working redox couple of the oxide and carbon coat. Polymers facilitate good electrical contact between the carbon coating on the oxide materials and the current collectors. Among the conductive polymers incorporated, PPy exhibits a better charge rate capability (120 mAh g^−1^ at 10 C) than the PANI incorporated materials (60 mAh g^−1^ at 10 C). It is also found that electrodeposited PPy on the C–LFP shows better performances (120 mAh g^−1^ at 10 C) than the chemically synthesized composite of PPy/C–LFP (70 mAh g^−1^ at 10 C) [110]. Andrea Fedorková’s group synthesized the PPy–LFP composite by the chemical oxidative polymerization of pyrrole monomer on the LFP particles’ surface [109]. In their study, they have utilized polyethylene glycol (PEG) as an additive during the polymerization that is the decrease the particle-to-particle contact resistance. Consequently, the PPy/PEG composite polymer decreases the charge transfer resistance of the LFP electrodes. The study emphasizes the thought that the combination of PEG with PPy gives the stabilizing effect that reduces the crosslinking and reduces the structural defects, causing the higher specific capacity for PPy/PEG composite utilization than the PPy only [111,112]. PPy/PEG–LFP exhibits the discharge capacity of 156 mAh g^−1^ in comparison to PPy–LFP of 147 mAh g^−1^ at a C/5 rate [109]. Gong et al. prepared the LFP/carbon/PPy composite cathode by the chemical vapor deposition method [113]. In the vapor phase deposition process, pyrrole gas can penetrate through the inner pores of the LFP, which helps to reduce the undesirable reactions with the electrolytes and would inhibit the Li intercalation. The study emphasizes the electrochemical performance at high temperature. PPy surrounding the particles improves the ionic conductivity and reduces the electrode resistance, as well as decreases the dissolution of Fe in the LiPF_6_ electrolyte and the reduction in Fe ion at the electrode at elevated temperature. The cathode of LFP/carbon/PPy shows excellent rate capability (80 mAh g^−1^ at 20 C rate) at 20 °C and good cycle stability at 55 °C, even after 700 cycles, retaining the discharge capacity of 110 mAh g^−1^, maintaining 82% of the initial capacity at 5 C [113]. The Gao group modified the LFP to nanorods by solvothermal methods, which shows excellent electrochemical properties [114]. Shortening of the crystal size of the LFP along the b–axis shortens the diffusion path of Li^+^ extraction/insertion. Then, LFP was uniformly coated with the PPy (5 nm thickness) by in situ chemical oxidative polymerization, which does not affect the structure and morphology of the nanorods of LFP and creates the three-dimensional network connections that facilitate the electron transport effectively. The LFP/PPy cathodes exhibit initial specific capacities of 153, 138, and 118 mAh g^−1^ at 0.1 C, 1 C, and 5 C, respectively. And the initial discharge capacities of 128, 106.5, and 85.7 mAh g^−1^, at 0.1 C, 0.5 C, and 1 C, respectively, at –20 °C [114]. The Amaral group synthesized the LFP/polyaniline composite to enhance the electrochemical properties of the LFP by a low temperature solvothermal method (200 °C) [115]. The conducting polymer of polyaniline bears high conductivity and creates the polarity difference between the cathode and electrolyte particles. This factor improves the electrolyte permeation on the active particles. Polyaniline forms the conducting network that connects the LFP particles and reduces the electrical resistivity among the particles. The synthesis process utilized the solvothermal method at low temperature, which provides the high crystallinity LFP without the secondary phase, and chemical interaction between the polyaniline and LFP provides low charge transfer resistance and intensifies the electrochemical properties.

### 3.3. Conductive Polymer in Spinel-Type Cathode

**Figure 14:**
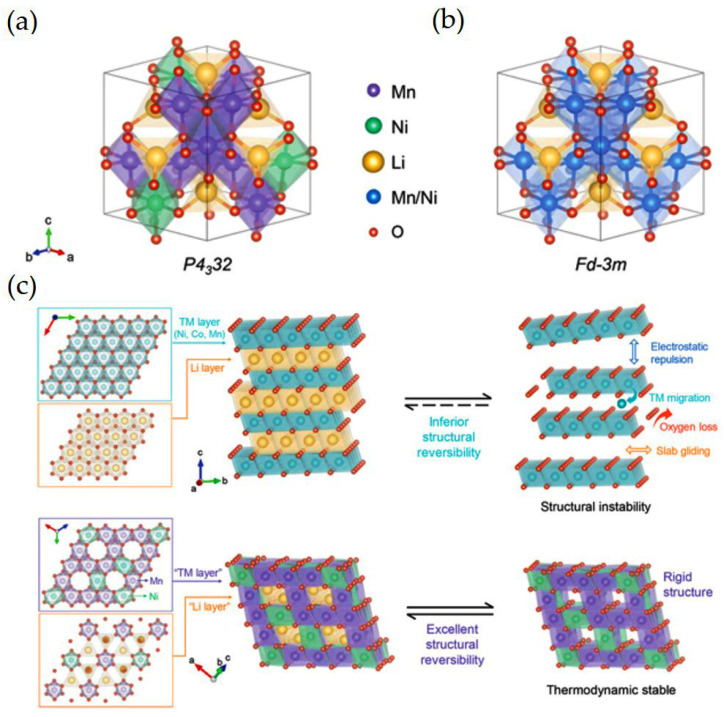
Crystal structures of (**a**) LNMO (P4332), (**b**) LNMO (*Fd3m*), and (**c**) comparison of spinel structure with Ni-based NCMs structure and electrochemical profile. Reproduced with permission. Reproduced with permission [122], copyright 2024, Advanced Materials published by Wiley-VCH GmbH.

Mn-rich, lithium manganese oxide (LiMn_2_O_4_, LMO, cubic spinel crystal structure) is prominent for higher energy layered oxide materials, high operation voltage window (4.0 V), high threshold on thermal runaway, and cost effectiveness [116]. It is also beneficial for supporting fast charging and discharging, and does not suffer from overcharging and thermal runaway. Various research focused on the morphological engineering to improve high-rate discharge capacity and capacity retention based on nanostructure tuning. Nano-size modification or tuning results in the longer cycle life of LMO materials [117,118,119]. The success is confined to cost-effectiveness, as nanoscaling is a costly process, hindering its practical implementation. Nickel-doped spinel LiNi_y_Mn_2-y_O_4_ (LNMO) is another prominent candidate of the spinel family for the next generation of LIBs due to its high-energy density, high operational voltage window (4.7 V), and higher threshold against thermal runaway [120]. It is considered that there are two possible space group structures of the LNMO. First, Ni replaces the part of Mn at the 16d site, forming (Ni, Mn)–O bonds (results in face-centered cubic structure, *Fd3m* space group) (Figure 14) [121,122,123]. Second, Ni replaces the part of Mn at the 4a position, and Mn occupies the 12d position, forming Ni–O and Mn–O bonds (results ordered cubic structure P4332 space group). In the disordered part (*Fd3m*), Li migrates from the 8a tetrahedral site to the 16c vacancy, denoted as 80–16c diffusion path. In the ordered region (P4332), 16c octahedral vacancies are distributed across octahedral 4a and 12d (1:3 ratio), giving the 8c–4a and 8c–12d diffusion path too. The three-diffusion path length Coulombic potential follows the order 8c–4a < 8a–16c < 8c–12d and follows the easiest to hardest diffusion path as well. The ordered P4332 structure is the most favorable diffusion path, bearing 25% of the total diffusion path, and ordered *Fd3m* is more favorable for the Li ion diffusion. Mn^3+^ in the disordered spinel is the contributor of the conductivity (10^−4.5^ S cm^−1^). In the ordered part, Ni/Mn ordering lowers the barrier to ionic mobility. Mn^4+^ stability is better than that of the Mn^3+^ octahedron. The transformation of the ferromagnetic Mn^4+^-O_2_-Mn^4+^ into the antimagnetic Ni^2+^-O_2_-Mn^4+^ increases the material’s structural stability. So, the thermal stability of the ordered structure is better than that of the disordered structure [124]. As a cathode material, spinel materials challenge the dissolution of transition metals, loss of active material, and increase in interfacial impedance [125,126]. The Jahn–Teller effect leads to partial rupture and microcracking, impacting the structural stability of the materials. The material’s surface structure is destroyed by complex side reactions of the material between the surface and the electrolyte, generating by-products that hinder ion transfer and result in the interfacial destabilization [121,127,128].

To enhance the stability and hinder undesirable interface reaction, and resist the dissolution amounts of the transition metal to the electrolyte protective layer coating improves the performance. Protective coatings of polymer (polyimide, polypyrrole) by thermal polymerization or chemical oxidation polymerization on the surface of the LMNO improve the cycling stability at room and elevated temperatures [129,130,131]. Nevertheless, lithium-ion storage requires more development in terms of stability and conductivity. Conductive polymer plays a crucial role in developing electrochemical performance. Dong et al. coated the spinel structure of LNMO with polyaniline (PANI), showing the capacity of 123 mAh g^−1^ and 99.7% capacity retention at 0.5 C after 200 cycles in the voltage range of 3.0–4.95 V (vs. Li/Li^+^) at room temperature [132]. The PANI acts as a coating layer to hinder the dissolution of the Ni and Mn from the LNMO cubic structure into the electrolyte, as well as enhance the electrical conductivity of the electrode, and removes the undesirable interfacial reactions between cathode materials and electrolytes. Mousa group utilized the long nanorods of LMO covered by the PANI layer as cathode materials [133]. The compactness of the PANI on the LMO surface is a strong coupling and more delocalization through the grain boundary. Higher conductive polymers bear a small diffusion path of the ions in the electrolyte and decrease internal resistance and enhance the electrolyte ion transport rate, which could improve the capacity. The composite materials exhibit higher electrical conductivity (5.5 × 10^−2^ S cm^−1^) than PANI (9.1 × 10^−4^ S cm^−1^). The composite materials also showed a higher capacity of 75 mAh g^−1^ at a scan rate of 5 mV s^−1^ in 1 M LiNO_3_ electrolyte compared with that of PANI (33 mAh g^−1^) and 82% capacity retention after 100 cycles. Another study, the Goswamy group designed the LMO–rGO/PANI nanocomposite as a cathode material for Li–ion batteries [134]. LMO nanocomposite was synthesized by the hydrothermal process, and a composite of LMO–rGO/PANI was prepared by the exfoliation of rGO and PANI on it. The addition of the rGO reduces the crystallite size of the LMO from 40 nm to 21 nm and improves electrical conductivity and the reduction in LMO aggregation. And PANI incorporation improves the stability and retention as well as the electrochemical performances. LMO–rGO/PANI exhibits the capacity of 135 mA h g^−1^ with an 85% retention after 100 cycles; on the other hand, LMO shows the capacity of 105 mAh g^−1^ with 70% of capacity retention after 100 cycles. Here, the incorporation of the rGO and PANI increases the electrical conductivity and reduces the structural aggregation, resulting in improved electrochemical performance [134].

**Figure 15:**
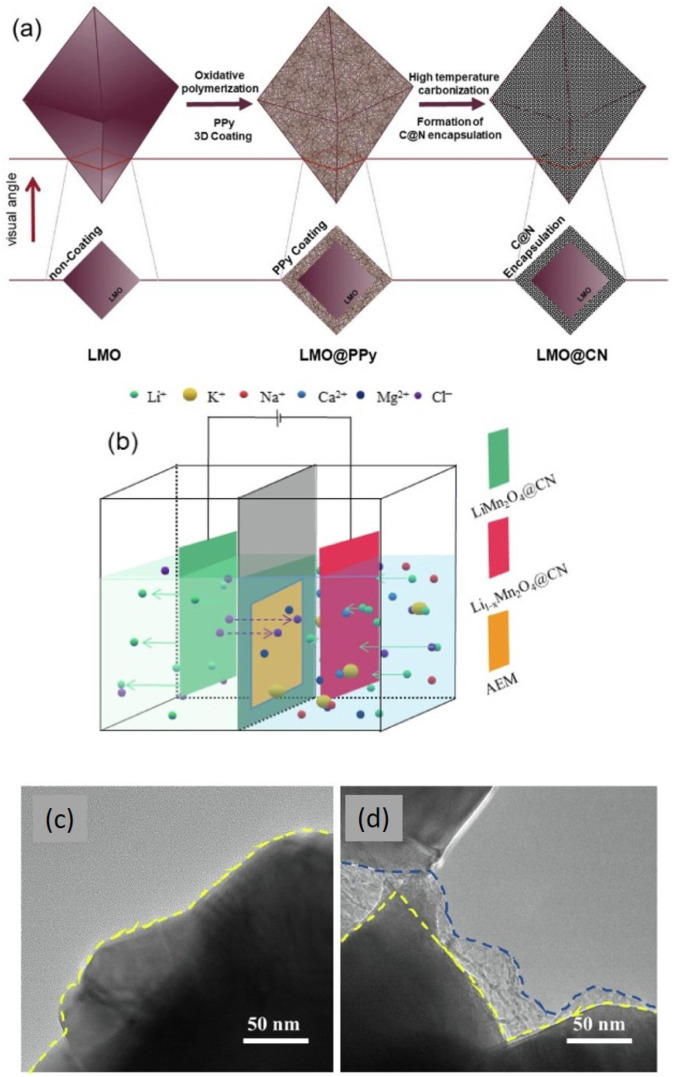
(**a**) Schematic of the materials synthesis, and (**b**) Li extraction process, TEM image of (**c**) LMO, and (**d**) N-doped carbon-coated (polypyrrole) LMO. Dashed line demonstrates the distinct coating layer. Reproduced with permission [135], copyright 2021, Elsevier.

**Figure 16:**
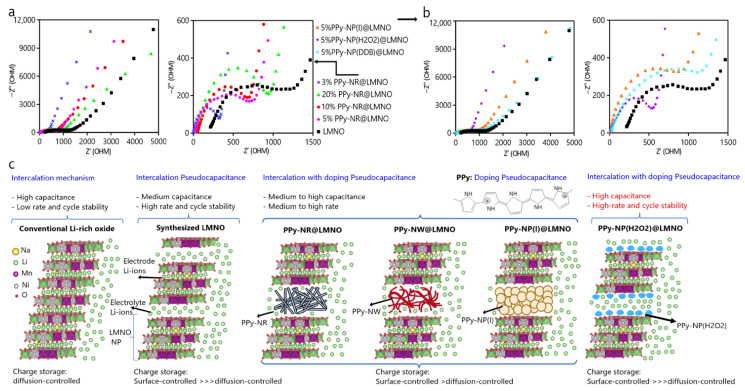
Nyquist plots of (**a**) LMNO and PPy-NR@LMNO electrodes, (**b**) LMNO and PPy-NP@LMNO electrodes without any cycle, and (**c**) schematic comparison of redox pseudocapacitance, intercalation pseudocapacitance, and intercalation with doping pseudocapacitance. Reproduced with permission [138], copyright 2022, Elsevier.

The Yuan group increases the electrosorption and cycle stability of the LMO cathode by surface modifications (Figure 15) [135]. The approach utilized the conductive polymer of polypropylene (PPy) as a precursor by in situ polymerization on the LMO surface to produce carbon encapsulation surrounding the LMO materials, where nitrogen is in uniform distribution. Electrodeposition rate and cycle stability are improved by nitrogen doping, encapsulation, and optimized crystal. The nitrogen-doped carbon encapsulated LMO electrode retains 80% capacity after 50 cycles at 1 C (145 mAh g^−1^) rate, which is 20% higher than bare LMO [135]. Another study by the Liu group in situ polymerization growth of PEDOT [poly(3,4-ethylenedioxythiophene)] by thiophene monomer (EDOT), with ammonium persulfate (APS) as oxidizing agent, p-toluenesulfonic acid (PTSA) as dopant on LMNO [136]. The approach improves the cycle performance of the LMNO cathode, where discharge capacity drop is slower (114 mAh g^−1^ after 100 cycles) than bare LNMO (98 mAh g^−1^ after 100 cycles). Here, the PEDOT layer improves the conductivity and suppresses the dissolution of the transition metal into the electrolyte, consequently improving the cycle life performance. The W. Zhu group proposes an innovative approach to the utilization of CePO_4_ as an ion conductor, on the LMO loaded with the crystalline CePO_4_ by the sol–gel process [137]. This enhances the stability of the LMO structure, and the redox reaction between Ce^4+^ and Ce^3+^ enhances the surface electrical conductivity and charge distribution of the LMO. Further enhancing the hydrophilicity of CePO_4_-loaded LMO by utilizing the polyacrylic hydrophilic binder improves the lithium extraction capacity. Here, the CePO_4_ loading optimizes the effective Li ion transport kinetics on the LMO surface, and hydrophilicity increases the Li^+^ active sites, resulting in lithium extraction capacity. Hydrophilicity of 1%CePO_4_ loaded LMO maintains the 83.8% discharge capacity after 30 cycles and maintains the optimal lithium-ion extraction/release capacity of 32.72 and 21.8 mg g^−1^ [137]. Voznyy group developed the LMNO cathode with functionalized polypyrrole (PPy) nanostructure coating (Figure 16) [138]. PPy bears the theoretical capacity of 72 mAh g^−1^ in the range of 2–4.5 V (vs. Li/Li^+^), considered to streamline the energy storage capacity and improve the capacity retention of the LMNO cathode. Types of nanocomposites, such as PPy as nanoparticles (PPy-NP), nanorods (PPy-NR), and nanowires (PPy-NW), with different weight percentages, shapes, sizes, and distribution quality, influence the electrochemical performance of the cathode. Synthesis process optimization and tuning provide the various shapes, sizes, and quality of distribution of nanoparticles in the cathode materials, resulting in different performance phenomena. It is found that 5% PPy-NP (H_2_O_2_)@LMO exhibits the efficient reversible capacity of 200 mAh g^−1^ and good cycle life and capacity retention of 96% after 200 cycles at 0.1 C. Modified electrodes by the PPy-NP (H_2_O_2_) and PPy–NP (DDB) show a smaller size and higher dispersity and homogeneity quality than among the different kinds of PPy-NP. It is quite evident that decreasing the dimensions and increasing the dispersity structure enhances the interfacial area due to nanoparticles in the cathode of LMNO, and as it increases the contact area of the electrolyte and decreases the diffusion length of the Li-ion. Surface-controlled processes separate diffusion and capacitive contribution and reduce the impedance of the SEI layer. Surfactant utilized to PPy-NP (H_2_O_2_) helps to distribute homogenous nanocomposites, giving efficient electrochemical performances [138]. In another study, the Wang group fabricated the PPy/LMO with Al_2_O_3_ as cathode materials [139]. Al_2_O_3_ coating improves the surface area of the active materials, resulting in the improvement of the stability and lithium storage capacity. PPy/Al_2_O_3_/LMO cathode shows the 121 mAh g^−1^ capacity at 1 C and 96% capacity retention after 100 cycles. Brilloni et al. synthesized the bifunctional electronically conductive poly(3,4-ethylenedioxythiophene)-poly(styrene sulfonate) (PEDOT: PSS) with LMNO as cathode materials. It is found that PEDOT: PSS acts as a barrier for the undesired side reaction of the LMNO [138]. It was demonstrated that almost 100% capacity retention was achieved over 200 cycles at a C/10 current rate with a high cut-off voltage of 5 V.

**Figure 17:**
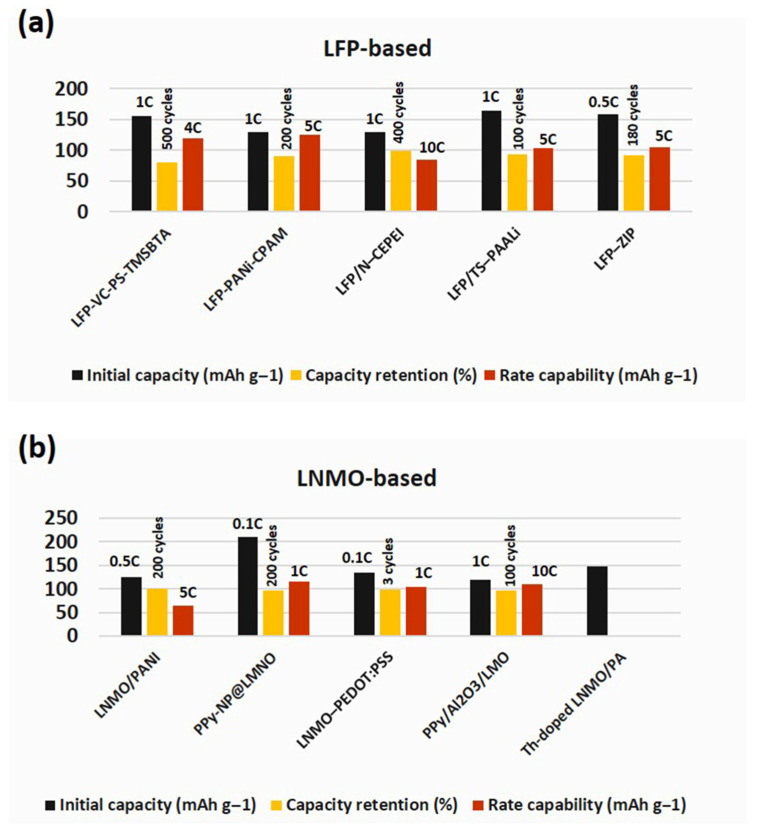
Comparison of the initial capacity, capacity retention, and rate capacity of different developed materials of (**a**) LFP-based, (**b**) LNMO-based.

Watanabe group utilized the LNMO with a hybridized gel polymer cathode (GPC) and gel polymer electrolyte (GPE) containing sulfone-based electrolyte for the application of the flexible lithium batteries for the wearable electronics [140]. The battery fabrication employs simple laminations with a metallic lithium anode. There is no need for the injection of electrolytes. GPC flexibility with a hierarchical 3D porous structure helps to form an ion-conducting path, and the GPE membrane has significant ionic conductivity for significant capacity output (115 mAh g^−1^) with excellent capacity retention (90%) under continuous deformation [140]. Lastly, to achieve high-performance and highly stable spinel LMO materials, in perspective of research trends, the focus needs to be on physicochemical properties of coating layers and structural criteria. Structural criteria should ensure the formation of a physical barrier, uniformity, and thinness. And physicochemical properties should ensure the high chemical stability, good ionic/electronic conductivity, strong mechanical adhesion, and ensure the resistance of electrolyte corrosion and Mn dissolution [141]. A comparison of the electrochemical performances of olivine and spinel-type electrode materials with and without polymer coatings is presented in Table 2 and Figure 17 (bar graph). The comparison shows substantial differences in performance metrics, such as charge storage capacity, cycling stability, and rate performance.

### 3.4. Conductive Polymer Selection and Design Consideration for the Cathode

Conductive polymers (CPs) such as PANI, PPy, PEDOT, and PAc can significantly improve the electronic conductivity, rate performance, and structural integrity of LIB cathodes but may also compromise specific capacity due to their inactive mass and limited electrochemical storage. Thus, it is critical to underline that appropriate polymer choice, doping, and interface engineering are essential to address these trade-offs, as represented in Table 3.

Conductive polymers such as PPy and PANI can undergo volumetric and structural changes during repeated cycling, which can adversely affect long-term cyclability if not carefully optimized. PEDOT usually demonstrates better electrochemical robustness but requires careful surface engineering to avoid interfacial degradation and side reactions. For instance, in PEDOT:PSS, acidic sulfonate groups of PSS can affect interfacial stability if not carefully managed. Uniform coating is a key factor for performance gains; incomplete coating can leave active particle surfaces unprotected, limiting benefits.

The failure modes for cathode performance degradation across layered, olivine, and spinel cathodes differ strongly by cathode family, so the “best” CP depends on the specific application and chemistry rather than being universal.

(a) Layered oxides (LCO/NCM/NCA): prioritize CEI stabilization and crack suppression at high voltage

Layered oxides are dominated by transition metal dissolution, high-voltage electrolyte oxidation, and microcrack-driven surface reconstruction, especially in Ni-rich systems. CPs that form uniform, ion-permeable, and conformal coatings are advantageous. For example, PEDOT:PSS is frequently used as an interfacial modifier because it provides an electronically conductive yet ionically permeable coating that reduces impedance growth and improves retention in layered systems. When the dominant issue is particle cracking and interfacial side reactions, PPy (especially as part of dual-layer coatings with inorganic protectors such as Li_3_PO_4_/LiAlO_2_/Li_2_SiO_3_) is highly suitable because the polymer can “seal” defects in inorganic layers while adding flexible electronic pathways, leading to notable improvements in capacity retention and high-rate performance for these dual-coating strategies. For Ni-rich NCM, PANI coatings are particularly relevant when the key problem is cation mixing/residual surface alkalinity and structural degradation, as shown by reduced cation mixing indicators and suppressed surface residues in PANI-coated NCM811.

(b) Olivine (LFP/LMFP): prioritize electron transport + HF/metal-dissolution tolerance without blocking Li^+^ pathways

Olivine cathodes are primarily limited by low intrinsic electronic conductivity and sluggish kinetics, while long-term operation can be impacted by HF-driven Fe dissolution and interfacial degradation. Accordingly, CP strategies that create continuous electronic pathways while maintaining porosity are preferred. Within the LFP literature summarized in this manuscript, PANI stands out as a strong candidate for improving high-rate performance when integrated with carbon-coated LFP. In addition, functional CP binders (e.g., carboxylated or zwitterionic systems) are attractive for olivine because adhesion/dispersion and ion-conduction in the electrode film can be as critical as surface coating chemistry. This aligns with the manuscript’s broader perspective that many CP–cathode composites underperform due to poor adhesion or uneven coatings, and that introducing polar functional groups (–OH/–COOH) and building crosslinked/stretchable networks can materially improve integrity.

(c) Spinel (LMO/LNMO): prioritize metal-dissolution suppression and oxidative stability

Spinel cathodes (especially LNMO) face significant challenges from Mn/Ni dissolution, surface parasitic reactions, and high-voltage instability, so the CP must be oxidatively robust and should not undergo rapid over-oxidation/degradation during cycling. The manuscript already emphasizes that chemically robust CP backbones (including crosslinked/dopant-optimized variants) are required to avoid overoxidation, oxidative degradation, and volumetric changes during electrochemical operation. Therefore, for high-voltage spinel cathodes, CP choices should lean toward oxidation-tolerant polymers (often including polyimide-type or stabilized CP formulations) and architectures that function as a protective yet Li^+^-permeable barrier, rather than thick electronic-only shells.

A rational selection should therefore start from the primary bottleneck (interfacial instability vs. bulk electronic transport vs. mechanical cracking vs. metal dissolution) and then choose CP chemistry, functional groups, and architecture accordingly. Importantly, CP design must balance electronic percolation with ion-transport accessibility, because dense/impermeable CP layers can improve electronic conductivity while hindering Li^+^ diffusion unless a porous/nanostructured network is used.

## 4. Scope of Improvement and Future Prospect

The functional roles of conductive polymers in cathode design for Li-Ion batteries have advanced significantly over the past few decades; there are still several areas that can be further improved. Conductive polymers are incorporated into cathodes to enhance their integrity while ensuring consistency in performance. To obtain the benefits of these factors, it is necessary to consider whether the applied conductive polymer falls within the suitable volume range, maintains the proper adhesion with intrinsic particles, and ensures chemical compatibility. Chemically robust CP backbones with crosslinked or dopant variants are required to develop materials that avoid overoxidation, oxidative degradation, and volume expansion during electrochemical.

CP composites with LIB cathodes suffer from poor adhesion or uneven coating. Most research works lack focus on tuning or engineering the adhesion properties of conductive polymers, despite the importance of understanding how long-term operation influences adhesion and maintains integrity. Based on the adhesive performance and fundamentals of intrinsic adhesion, the nature of the polymer can be modified by optimizing the number of hydroxyl and carboxyl groups, and also examining how these modifications affect long-term operational performance. Epoxy- and phenoxy-based materials have potential and provide solid adhesion. Conductive polymer hybridization with shorter molecular weight of phenoxy- or epoxy-based materials could provide a significant impact on the development of efficient cathode materials for long-term operations. Various temperature ranges of materials need to be considered significantly, which is overlooked by research groups. A conductive polymer maintains efficient conductivity in moderate temperatures, but at higher temperatures, phase transformation or separation may occur, deteriorating the long-cycle operation. Therefore, it is required to evaluate the high-temperature stability of the polymeric materials for a proper electrochemical operation.

It is well known that composite cathode electronic conductivity is enhanced by CPs, but it may hinder ionic diffusion if not sufficiently porous or structured. Hierarchically porous or nanostructured CP networks facilitate ion transport while preserving a percolated electron pathway. The redox-active CPs (e.g., quinone-based or hybrid redox polymers) that actively participate in charge storage provide dual benefits of conductivity and capacity enhancement. Scalability of synthesis methods is a crucial aspect that must be considered. Although methods such as electrochemical deposition, chemical oxidative polymerization, or vapor phase polymerization have shown promise, they are usually not suitable for large-scale commercial production. Therefore, special attention should be directed toward scalable synthesis methods, such as UV-curing, 3D printing, or water-based dispersion techniques for CP coating.

In situ operando evaluation needs to be considered, as it presents the idea of operation optimization for the materials design. Conductive polymers help accommodate volume expansion and shrinkage. This approach could provide strategic insights for optimizing the design of accommodation space to ensure strong contact between the active materials. Furthermore, it is crucial to give considerable thought to the design of the conductive polymer with a stretchable network, as these characteristics will aid in preserving structural integrity. Moreover, it is important to develop and focus studies on the numerical simulation of electrochemical operation. Understanding the fluid dynamics of in situ operation can help achieve precise control over electrochemical behavior and enhance performance by designing materials with the desired mechanical, physicochemical, and structural properties. Structural studies criteria help to understand the formation of a physical barrier, uniformity, and thinness. Physicochemical properties studies will provide the knowledge of chemical stability, ionic/electronic conductivity, adhesion nature, and resistance to dissolution and corrosion.

## Figures and Tables

**Table 1:** Comparison of the electrochemical performance of various layered cathode materials in Li-ion batteries, with or without conductive polymer coating.

Materials	Electrochemical Performance			Ref.
	Initial Capacity @Current Rate	Capacity Retention	Rate Capability @Current Rate	
LiCoO_2_/PPy	~182 mAh g^−1^ @0.2C	94.3% (200th cycles)	~146 mAh g^−1^ @10C	[63]
LiCoO_2_	~189 mAh g^−1^ @0.2C	83.5% (200th cycles)	~98.7 mAh g^−1^ @10C	
NCM811/PANI–PVP	~202 mAh g^−1^ @200 mA g^−1^	88.7% (100th cycles)	~152 mAh g^−1^ @1000 mA g^−1^	[71]
NCM811	~198 mAh g^−1^ @200 mA g^−1^	66.3% (100th cycles)	~111 mAh g^−1^ @1000 mA g^−1^	
V-NCA/PEDOT:PSS	~183.3 mAh g^−1^ @1C	83.5% (at 200th cycles)	~157.7 mAh g^−1^ @5C	[77]
V-NCA	~179.2 mAh g^−1^ @1C	66.7% (200th cycles)	~148.7 mAh g^−1^ @5C	
NCM/PPy-LiAlO_2_	~189.1 mAh g^−1^ @0.1C	92.8% (1C at 100th cycles)	~128.0 mAh g^−1^ @10C	[65]
NCM/PPy	~182.0 mAh g^−1^ @0.1C	90.9% (1C at 100th cycles	~118.0 mAh g^−1^ @10C	
NCM	~192.0 mAh g^−1^ @0.1C	83.0% (1C at 100th cycles)	~86.0 mAh g^−1^ @10C	
NCM/PPy-Li_3_PO_4_	~203.0 mAh g^−1^ @0.1C	86.5% (1C at 200th cycles)	~159.7 mAh g^−1^ @10C	[66]
NCM-PPy	~204.5 mAh g^−1^ @0.1C	76.1% (1C at 200th cycles)	~138.6 mAh g^−1^ @10C	
NCM	~206.0 mAh g^−1^ @0.1C	65.8% (1C at 200th cycles)	~125.7 mAh g^−1^ @10C	
NCM/PPy-Li_2_SiO_3_	~218.8 mAh g^−1^ @0.05C	96.4% (10C at 100th cycles)	~145.1 mAh g^−1^ @10C	[69]
NCM-Li_2_SiO_3_	~213.0 mAh g^−1^ @0.05C	87.5% (10C at 100th cycles)	~108.4 mAh g^−1^ @10C	
NCM	~204.7 mAh g^−1^ @0.05C	84.5% (10C at 100th cycles)	~82.2 mAh g^−1^ @10C	
NCM/PPy-Li_3_VO_4_	~185.6 mAh g^−1^ @0.5C	93.7% (0.5C at 100th cycles)	~139.5 mAh g^−1^ @10C	[70]
NCM-PPy	~183.8 mAh g^−1^ @0.5C	78.9% (0.5C at 100th cycles)	~119.0 mAh g^−1^ @10C	
NCM	~184.8 mAh g^−1^ @0.5C	73.6% (0.5C at 100th cycles)	~89.5 mAh g^−1^ @10C	
NCM/PANI	~218.8 mAh g^−1^ @0.05C	96.2% (1C at 80th cycles)	~141 mAh g^−1^ @5C	[72]
NCM	~224.9 mAh g^−1^ @0.05C	90.0% (1C at 80th cycles)	~128 mAh g^−1^ @5C	
NCM/PPy	Diffusion coefficient~8.4 × 10^−10^ cm^2^ s^−1^	90.7% (1C at 100th cycles)	~136 mAh g^−1^ @10C	[64]
NCM	Diffusion coefficient~1.1 × 10^−10^ cm^2^ s^−1^	78.8% (1C at 100th cycles)	~76 mAh g^−1^ @10C	
NCM@PANI	~221.0 mAh g^−1^ @0.3C	84.0% (0.1C at 100th cycles)	~174.5 mAh g^−1^ @1C	[74]
NCM	~204.0 mAh g^−1^ @0.3C	56.0% (0.1C at 100th cycles)	~121.5 mAh g^−1^ @1C	
LNM95/PPy	~150.9 mAh g^−1^ @5C	90.1% (1C at 100th cycles)	~150.9 mAh g^−1^ @5C	[59]
LNM95	~105.8 mAh g^−1^ @5C	66.7% (1C at 100th cycles)	~105.8 mAh g^−1^ @5C	
NCM811/EPS	~176.1 mAh g^−1^ @1C	95.6% (1C at 100th cycles)	~144.6 mAh g^−1^ @5C	[75]
NCM811	~182.8 mAh g^−1^ @1C	57.7% (1C at 100th cycles)	~133.3 mAh g^−1^ @5C	
NCM/*Psi	~191.1 mAh g^−1^ @1C	91.5% (1C at 120th cycles)	~171.4 mAh g^−1^ @5C	[76]
NCM	~192.8 mAh g^−1^ @1C	71.4% (1C at 120th cycles)	~148.8 mAh g^−1^ @5C	
NCM/Polyethylene	~190.3 mAh g^−1^ @1C	82.9% (1C at 200th cycles)	~170.5 mAh g^−1^ @5C	[78]
NCM	~183.8 mAh g^−1^ @1C	53.9% (1C at 200th cycles)	~175.5 mAh g^−1^ @5C	
NCM@PANI-PEG	~201.6 mAh g^−1^ @0.2C (at 25 °C)	93.4%@1C (at 25 °C); 81.4%@1C (at 55 °C) at 100th cycles	~156.7 mAh g^−1^ @10C	[73]
NCM@PANI	~199.4 mAh g^−1^ @0.2C (at 25 °C)	89.6% (1C at 25 °C); 77.1% (1C at 55 °C) at 100th cycles	~151.0 mAh g^−1^ @10C	
NCM	~194.7 mAh g^−1^ @1C (at 25 °C)	83.4% (1C at 25 °C); 53.6% (1C at 55 °C) at 100th cycles	~139.4 mAh g^−1^ @10C	
NMT96@PI/PVP	~192.3 mAh g^−1^ @C/3	86.7% @C/3 (1C = 1.5 mA cm^−2^) at 500th cycles	~171.4 mAh g^−1^ @5C	[79]
NMT96	~193.8 mAh g^−1^ @ C/3	63.2% @C/3 (1C = 1.5 mA cm^−2^) at 500th cycles	~148.8 mAh g^−1^ @5C	
NCM622/*cPAN	~182.3 mAh g^−1^ @ C/3	88.6% (1C at 200th cycles)	~156.6 mAh g^−1^ @5C	[80]
NCM622	~179.7 mAh g^−1^ @ 0.1C	73.2% (1C at 200th cycles)	~140.0 mAh g^−1^ @5C	
NCM/LiMTFSI	~156.4 mAh g^−1^ @ 4C	81.8% (4C at 200th cycles)	~103.08 mAh g^−1^ @8C	[81]
NCM	~159.6 mAh g^−1^ @ 4C	49.7% (4C at 200th cycles)	~81.54 mAh g^−1^ @8C	
NCM/PEDOT	~194.0 mAh g^−1^ @ 4C	91.93% (1C at 100th cycles)	~143.8 mAh g^−1^ @5C	[86]
NCM	~187.0 mAh g^−1^ @ 1C	79.92% (1C at 100th cycles)	~130.0 mAh g^−1^ @5C	
LCoO_2_@PAN	~182.0 mAh g^−1^ @0.7C	93.2% (0.7C at 300th cycles)	-----	[87]
LCoO_2_	~185.0 mAh g^−1^ @0.7C	87.3% (0.7C at 300th cycles)	------	
NCM111/Polyimide	~180.0 mAh g^−1^ @1C	52.0% at 300th cycles	~140.0 mAh g^−1^ @5C	[88]
NCM111	~181.0 mAh g^−1^ @1C	66.0% at 300th cycles	~145.0 mAh g^−1^ @5C	

* Psi = Polysiloxane; cPAN = Cyclized Polyacrylonitrile

**Table 2:** Electrochemical performance comparison of various cathode materials in Li-ion batteries with or without conductive polymer.

Materials	Electrochemical Performance			Ref.
	Initial Capacity @Current Rate	Capacity Retention	Rate Capability @Current Rate	
LFP-VC-PS-TMSBTA	~155 mAh g^−1^ @1C	80.8% (500th cycles)	~120 mAh g^−1^ @4C	[102]
LFP	~140 mAh g^−1^ @1C	67.0% (500th cycles)	~110 mAh g^−1^ @4C	
LFP-PANi-CPAM	~130 mAh g^−1^ @1C	90.0% (200th cycles)	~125 mAh g^−1^ @5C	[103]
LFP–PVdF	~125 mAh g^−1^ @1C	83.0% (200th cycles)	~95 mAh g^−1^ @5C	
LFP/N–CEPEI	~130 mAh g^−1^ @1C	98.4% (400th cycles)	~85 mAh g^−1^ @10C	[104]
LFP–PVdF	~127 mAh g^−1^ @1C	86.6% (400th cycles)	~60 mAh g^−1^ @10C	
LFP/TS–PAALi	~165 mAh g^−1^ @1C	93.7% (100th cycles)	~103 mAh g^−1^ @5C	[94]
LFP-PVdF	~167 mAh g^−1^ @1C	89.8% (100th cycles)	~40 mAh g^−1^ @5C	
LFP–ZIP	~158 mAh g^−1^ @1C	91.7% (180th cycles)	~105 mAh g^−1^ @5C	[108]
LFP–PVdF	~155 mAh g^−1^ @1C	83.2% (180th cycles)	~90 mAh g^−1^ @5C	
LNMO/PANI	~125 mAh g^−1^ @0.5C	99.7% (200th cycles)	~65 mAh g^−1^ @5C	[132]
LNMO	~118 mAh g^−1^ @0.5C	96.0% (200th cycles)	~50 mAh g^−1^ @5C	
N–doped–C/PPy–LMO	~120 mAh g^−1^ @1C	87.5% (50th cycles)	–	[135]
LMO	~95 mAh g^−1^ @1C	73.6% (50th cycles)	–	
LNMO/PEDOT	~130 mAh g^−1^ @0.2C	91.2% (100th cycles)	–	[136]
LNMO	~125 mAh g^−1^ @0.2C	76.0% (100th cycles)	–	
LMO/CePO_4_/PAA	~120 mAh g^−1^ @1C	83.8% (30th cycles)	–	[137]
LMO	~100 mAh g^−1^ @1C	68.0% (30th cycles)	–	
PPy-NP@LMNO	~210 mAh g^−1^ @0.1C	96.0% (200th cycles)	115 mAh g^−1^ @1C	[138]
LNMO	~160 mAh g^−1^ @0.1C	96.8% (200th cycles)	90 mAh g^−1^ @1C	
PPy/Al_2_O_3_/LMO	~120 mAh g^−1^ @1C	96.0% (100th cycles)	110 mAh g^−1^ @10C	[139]
LMO	~97 mAh g^−1^ @1C	68.0% (100th cycles)	70 mAh g^−1^ @10C	
Th-doped LNMO/PA	~148 mAh g^−1^	–	–	[142]
LNMO	~105 mAh g^−1^	–	–	
LNMO–PEDOT:PSS	~135 mAh g^−1^ @0.2C	99.0% (3 cycles)	105 mAh g^−1^ @1C	[143]
LNMO–PVdF	~110 mAh g^−1^ @0.2C	97.6% (3 cycles)	25 mAh g^−1^ @1C	

**Table 3:** Advantages and disadvantages of key conductive polymers with composite cathode.

Conductive Polymer	Key Advantages/Aspects	Key Disadvantages/Drawback	Example
Polypyrrole (PPy)	- Strong mechanical strength and good intrinsic conductivity - Easy and scalable chemical polymerization - Boosts charge transport and mechanical flexibility	- Introduce inactive mass decreasing theoretical capacity - Poor electrochemical stability at high potential - Under extended cycling, swelling and conductivity fading may occur	- PPy-NCM cathode demonstrated improved capacity retention and rate performance, but solely PPy coating cannot completely stabilize the cathode during long-term cycling [66]. - PPy coating alone does not fully suppress the capacity fade of the LNMO cathode at high voltage and prolonged cycles [60]
Polyaniline (PANI)	- Improve electrical conductivity - Easy synthesis, compatible with sol–gel and self-assembling coating technique - Pseudocapacitive capacity contribution enhances rate capability - Enhances Li^+^ diffusion	- Add inactive mass in cathode composite, reducing specific capacity - Volumetric changes and degradation of the CP layer limit long-term cycling stability	- PANI-NCM composite showed a higher rate capability but decreased initial capacity [72]. - PANI cathode for LIBs delivered an initial specific capacity of 159.83 mAh g^−1^ with 75% capacity retention after 100 cycles [144].
PEDOT/PEDOT: PSS	- Excellent film-forming properties and high conductivity - Enhances rate performance and cycling stability - Stable electrochemical behavior within battery potential window	- Acidic character of PSS may corrode the cathode surface - Polymer layer introduces inactive mass, reducing net capacity - Possible interfacial resistance if the polymer is too thick	- PEDOT:PSS coatings have shown notable improvement in cycling stability for high voltage LNMO cathode, yet require optimization to mitigate PSS-induced corrosion [145]
Polyacetylene (PAc)	- Very high intrinsic conductivity (~105 S cm^−1^) when doped - Strong π-conjugated structure supports charge delocalization	- Extreme environmental instability-easily degrades in air or oxidizes - Doping and adhesion are technically difficult, so rarely used	- EIS data indicate that crosslinked PAN coating is more effective in depressing the growth of charge transfer resistance in the LIBs cathode [80,87]

## Data Availability

No new data were created in this study. Data sharing is not applicable to this article.

## Abbreviations

The following abbreviations are used in this manuscript:

TMSBTA	1-(trimethylsilyl)- 1H-benzotriazole
PEDOT	poly(3,4-Ethylenedioxythiophene)
PSS	poly(styrene sulfonate)
PEG	Polyethylene glycol
EDOT	3,4-ethylenedioxythiophene
COF	Covalent organic framework
LDH	layered-double hydroxide
N–CEPEI	N-Ethyl carboxylic acid functionalized polyethyleneimine
CPAM	cationic polyacrylamide
CE	Coulombic efficiency
ICP-MS	Inductively coupled plasma mass spectrometry
LFP	LiFePO_4_
LMO	LiMn_2_O_4_
LNMO	LiNi_y_Mn_2-y_O_4_
NMT	LiNi_0.96_Mg_0.02_Ti_0.02_O_2_
PPy	polypyrrole
PANI	polyaniline
LiMTFSI	Sulfonyl(trifluoromethane sulfonyl)imide methacrylate
PS	1,3-propane sultone
VC	vinylene carbonate
ZIP	zwitterionic polymer
Th	thorium
